# The *IGF1 *small dog haplotype is derived from Middle Eastern grey wolves

**DOI:** 10.1186/1741-7007-8-16

**Published:** 2010-02-24

**Authors:** Melissa M Gray, Nathan B Sutter, Elaine A Ostrander, Robert K Wayne

**Affiliations:** 1 Department of Ecology and Evolutionary Biology, University of California, Los Angeles, CA, USA; 2 Current Address: Laboratory of Genetics, University of Wisconsin, Madison, WI, USA; 3 Department of Clinical Sciences, College of Veterinary Medicine, Cornell University, Ithaca, NY, USA; 4 Cancer Genetics Branch, National Human Genome Research Institute, National Institutes of Health, Bethesda, MD, USA

## Abstract

**Background:**

A selective sweep containing the insulin-like growth factor 1 (*IGF1*) gene is associated with size variation in domestic dogs. Intron 2 of *IGF1 *contains a SINE element and single nucleotide polymorphism (SNP) found in all small dog breeds that is almost entirely absent from large breeds. In this study, we surveyed a large sample of grey wolf populations to better understand the ancestral pattern of variation at *IGF1 *with a particular focus on the distribution of the small dog haplotype and its relationship to the origin of the dog.

**Results:**

We present DNA sequence data that confirms the absence of the derived small SNP allele in the intron 2 region of *IGF1 *in a large sample of grey wolves and further establishes the absence of a small dog associated SINE element in all wild canids and most large dog breeds. Grey wolf haplotypes from the Middle East have higher nucleotide diversity suggesting an origin there. Additionally, PCA and phylogenetic analyses suggests a closer kinship of the small domestic dog *IGF1 *haplotype with those from Middle Eastern grey wolves.

**Conclusions:**

The absence of both the SINE element and SNP allele in grey wolves suggests that the mutation for small body size post-dates the domestication of dogs. However, because all small dogs possess these diagnostic mutations, the mutations likely arose early in the history of domestic dogs. Our results show that the small dog haplotype is closely related to those in Middle Eastern wolves and is consistent with an ancient origin of the small dog haplotype there. Thus, in concordance with past archeological studies, our molecular analysis is consistent with the early evolution of small size in dogs from the Middle East.

See associated opinion by Driscoll and Macdonald: http://jbiol.com/content/9/2/10

## Background

Domestic dogs exhibit a tremendous amount of phenotypic diversity in coat colour, skeletal proportion, and behaviour [1-3]. Understanding the underlying causes of this diversity has been a prime motivation for studies on the evolutionary history of domestic dogs and the genetic basis for phenotypic traits. The common ancestor of the domestic dog is the grey wolf [4-6]. However, molecular genetic evidence suggests that there were multiple domestication and/or interbreeding events between domestic dogs and grey wolves [7-10]. The timing and location of the dog's origin remains uncertain. Mitochondrial DNA sequencing studies suggest an East Asian origin with dates ranging from ~5000 to 16,000 years ago [4-6,11]. In contrast, archaeological studies suggest a Middle Eastern, Western Russian or European origin approximately 14,000-31,000 years ago [12-15].

In order to understand the molecular mechanism by which domestic dogs have rapidly diversified in body size, we previously identified a haplotype shared by small dogs that underlies the major-effect quantitative trait locus for size on dog chromosome 15 [16]. A ~75 kb selectively swept haplotype was found spanning the promoter, exon and introns of the *IGF1 *gene and was strongly associated with skeletal size. Fine-mapping demonstrated that a ~10 kb interval spanning intron 2 is most strongly associated with size variation. A short interspersed element SINEC_Cf integration and single nucleotide polymorphism (SNP) are in perfect linkage disequilibrium (LD) in this interval. These markers are fixed in the majority of small distantly related dog breeds which suggests that small size evolved early in the history of domestication. Our previous study showed that, with the exception of a few giant breeds (mastiffs, bullmastiffs, and rottweilers, for example), the derived 'small' SNP allele was rarely observed in large dog breeds [16]. Interestingly, the small size-associated haplotype was not observed in any wild canid surveyed, ranging from grey wolf (*Canis lupus*) to island fox (*Urocyon littoralis*). However, no extensive characterization of variation across *IGF1 *in grey wolf, the wild progenitor of domestic dogs, was done. This characterization is critical to the understanding of the evolutionary history of the *IGF1 *gene and its role in the history of domestic dogs. In this study, we survey a variety of grey wolf populations to better understand the ancestral pattern of variation at *IGF1 *with a particular focus on the distribution of the small dog haplotype and its relationship to the origin of the dog.

## Results

### Microsatellite, SNP and SINE (short interspersed elements) markers within *IGF1*

**Figure 1:**
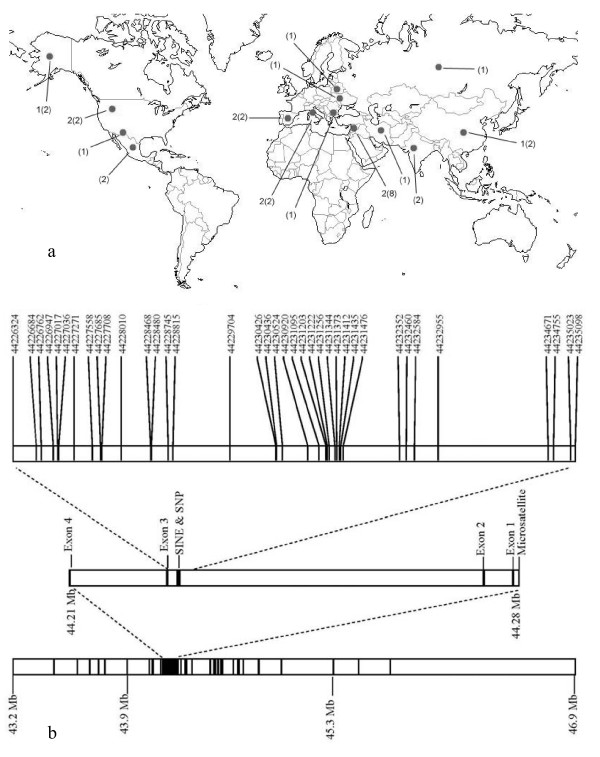
**Sample map of grey wolf populations sequenced and schematic of insulin-like growth factor 1 (*IGF1*) molecular markers and exons**. (a) The numbers outside the parentheses are sample sizes for the long sequence (6331 bps) and the numbers inside are sample sizes for the short sequence (4881 bps). (b) The chromosomal segment on the bottom shows the location of 94 dog-derived single nucleotide polymorphism (SNP) loci shown as black vertical lines. The chromosomal segments on the top show the locations of SNPs and indels discovered from sequencing.

**Figure 2:**
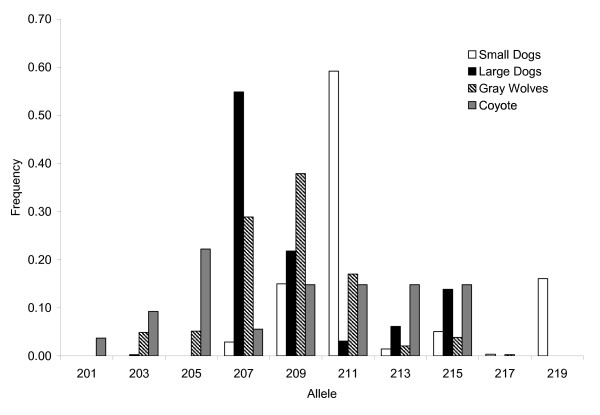
**Insulin-like growth factor 1 (*IGF1*) microsatellite allele frequency distributions in domestic and wild canids**. Allele sizes of the di-nucleotide repeats are given on the *X *axis and their frequency is on the *Y *axis.

The microsatellite located within the promoter region of *IGF1 *(CA_n_; CanFam1 44283699-44283736; Figure 1) displayed a significant association with body size in the domestic dog (*P *< 2.2 × 10-14, chi-square test) [16]. Specifically, the 207 base pair (bp) allele is associated with large sized and the 211 bp allele is associated with small sized domestic dogs. The microsatellite is in strong linkage disequilibrium with the SINE element and diagnostic SNP [16]. Although microsatellite alleles found in grey wolf spanned the entire range observed in dogs, the 209 bp allele, which is intermediate in length to the two alleles associated with body size in dogs, was found to have the highest frequency (41%) in 388 grey wolves worldwide (Figure 2, Additional File 1, Table S1). The completely overlapping allele sizes of grey wolves and small and large domestic dogs suggest this locus is not the causal mutation for small body size in dogs.

DNA sequence data confirmed the observation that no grey wolf had the diagnostic SNP allele (CanFam1 44228468; Figure 1) found in the intron 2 region of *IGF1 *in small dogs. Furthermore, we genotyped 374 grey wolves from 17 populations and 115 individuals from five distantly related wild canids (Additional File 1: Table S1) and found that no wild canid possessed the SINE element. The retrotransposon insertion and the diagnostic SNP allele appear to be unique to small domestic dogs. Therefore, these markers evolved uniquely in the domestic dog and are unlikely to have been segregating in the wolf ancestors of dogs.

### SNP Genotypes

**Figure 3:**
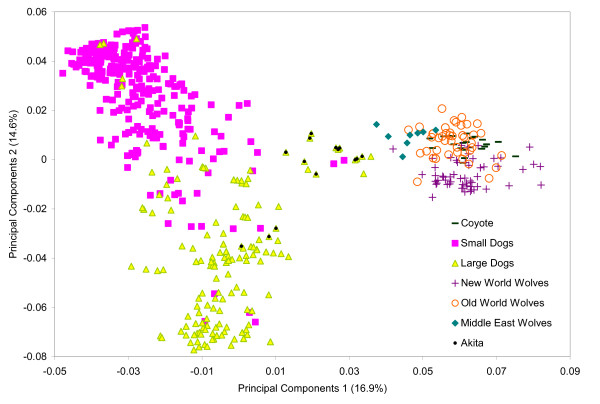
**Principal components analysis of 94 dog-derived single nucleotide polymorphism loci in domestic and wild canids**. Principal components one (PC1) is on the *X *axis and principal components two (PC2) is on the *Y *axis. The percent variation explained by each axis is also provided.

In order to investigate the evolutionary history of the *IGF1 *locus and the origin of the haplotypes found in domestic dogs, we performed principal components analysis (PCA) on genotypes from 94 dog-derived SNP markers spanning the *IGF1 *interval (Figure 1 and Additional File 1: Table S1 and S2; see Methods). Consistent with species level classification, domestic dogs were distinct from grey wolves and coyotes on the first PCA axis (Figure 3). On the second PCA axis, we observed separation between small and large domestic dogs and to a lesser extent between New World and Old World grey wolves. Furthermore, grey wolves of Middle East origin were slightly closer to domestic dogs than other grey wolf populations on the first PCA axis. Several Akita individuals, which is an ancient domestic dog breed [17], were positioned between grey wolves and the main cluster of domestic dogs (Figure 3). Outliers such as large bodied Rottweiler and mastiff dog breeds were observed within the small dog cluster. These breeds were previously found to have unexpected genetic similarity to small dogs in the *IGF1 *region [16]. The Boston terrier, which is the largest breed in our 'small dog' category, was the breed most associated with the large breed cluster. However, a few individuals from other small breeds were found there as well: cavalier King Charles spaniel, Chihuahua, toy fox terrier, miniature schnauzer, Norwich terrier and Shih Tzu. Moreover, all except the Chihuahua were previously found to exhibit some genetic similarity with large domestic dogs across the *IGF1 *locus [16]. Phylogenetic analysis of the SNP data defined a domestic dog cluster distinct from grey wolves (Figure S1). Further, the majority of small and large domestic dogs grouped separately from one another. No further resolution within each species was observed.

Individuals homozygous for the four most common of the *IGF1 *20 SNP marker haplotypes (B, C, F and I; 'Sutter haplotypes'; Additional File 2) were compared to homologous grey wolf and coyote haplotypes. However, haplotypes for only 17 of the 20 SNPs could be compared due to non-amplification of three SNPs in grey wolves. The wolf haplotypes most similar to the common small dog haplotype B are found in Spanish (haplotypes 22, 29, 47, 48 and 50) and Israeli (haplotype 15) wolves and differ from haplotype B by 3-4 substitutions (Additional File 2). However, such a comparison is intrinsically limited, since these SNPs were ascertained in a restricted panel of domestic dogs. Therefore, grey wolves may have additional variation that is undetected when using domestic dogs as a reference that could be informative for understanding the evolutionary history of the *IGF1 *gene. Nonetheless, this result supports the hypothesis that Middle East and Spanish wolves have haplotypes closest to those of small dogs.

### Long Sequence (6331 bp)

In order to further characterize *IGF1 *variation across grey wolf populations, we sequenced 6331 base pairs (bp) of intron 2 that directly surrounded the diagnostic 'small' SNP locus and the SINE element (Figure 1 and Additional File 1: Table S1). Fourteen phased haplotypes were identified from 30 SNPs and four indels (Additional File 3). We identified 13 private alleles in grey wolves and 20 polymorphic loci. In the coyote, we identified 12 private alleles and 11 polymorphic loci. Domestic dogs exhibited two private alleles and two polymorphic loci. However, these individuals were selected to be homozygous for the common and minor SNP haplotypes (see methods). In this analysis, grey wolf haplotypes from Israel (haplotypes 11 and 13) were most similar to haplotypes in small domestic dogs and differed from the common small dog haplotype 5 by 3-4 substitutions (Table 1 and Additional File 3). The presence of the SINE element and 'small' SNP allele in domestic dogs was responsible for two of the observed differences. A minimum spanning network constructed from pairwise nucleotide differences also exhibited a close relationship between the small dog haplotypes (Hap 5 and Hap 3) and haplotypes from Israel wolves (Hap 11, 13, and 14; Figure S2). Two haplotypes (Hap 4 and Hap 6) were shared between domestic dogs and grey wolves (Table 1). One Chinese and two Italian grey wolves were homozygous for Hap 4, which was shared with great Danes and Saint Bernards. One Alaskan and two Yellowstone grey wolf individuals possessed Hap 6 which was shared with Shih Tzu and mastiff. Nucleotide diversity was highest in coyote (0.00116, standard deviation [SD] 0.00082) followed by European grey wolves (Italy and Spain; 0.00054, SD 0.00035), and Israeli grey wolves (0.00047, SD 0.00037; Table 2).

**Table 1:** Matrix of single nucleotide polymorphism differences between haplotypes (Hap) from 6331 bp of sequence

	Hap1	Hap2	Hap3	Hap4	Hap5	Hap6	Hap7	Hap8	Hap9	Hap10	Hap11	Hap12	Hap13	Hap14
Hap1														
Hap2	11													
**Hap3 (HapC)**	14	9												
Hap4	13	8	3											
**Hap5 (HapB)**	13	8	5	8										
Hap6	14	9	4	1	9									
Hap7	15	10	5	2	10	1								
Hap8	19	14	9	6	14	7	8							
Hap9	11	6	7	4	6	5	6	10						
Hap10	15	10	5	2	10	1	2	8	6					
Hap11	13	8	9	8	**4**	9	10	14	4	10				
Hap12	16	11	6	3	11	2	3	9	7	3	11			
Hap13	12	7	8	7	**3**	8	9	13	3	9	1	10		
Hap14	14	9	10	9	**5**	10	11	15	5	11	3	12	2	

Bold haplotypes indicate small dog haplotypes. Bold numbers indicate haplotypes with smallest number of differences to the common small dog haplotype B.

**Table 2:** Nucleotide diversity estimates.

	6331 bps			4811 bps		
		
Populations	π	SD	n*	π	SD	n*
Small dog	0.00042	0.00030	3 (2)	0.00055	0.00040	5 (3)
Large dogs	0.00014	0.00012	5 (3)	0.00022	0.00018	5 (4)
North America	0.00016	0.00014	3 (2)	0.00043	0.00029	7 (6)
Europe	0.00054	0.00035	4 (3)	0.00044	0.00029	8 (6)
China	0.00000	0.00000	1 (1)	0.00010	0.00013	2 (2)
Middle East				0.00096	0.00055	11 (8)
Israel	0.00047	0.00037	2 (4)	0.00101	0.00058	8 (8)
Israel† (sampled)				0.00055	0.00044	2
India				0.00083	0.00062	2 (2)
Iran				0.00166	0.00176	1 (2)
Coyote	0.00116	0.00082	2 (2)	0.00111	0.00081	2 (2)

*The numbers of individuals (*n*) are given for each group with number of haplotypes in parentheses.

† Estimate is the median value of 1000 samples of two individuals from a sample of eight individual.

SD = standard deviation

**Figure 4:**
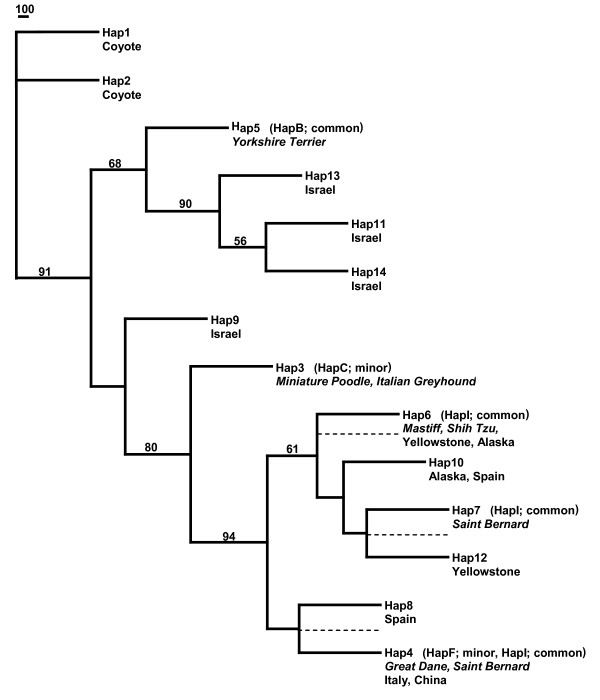
**Insulin-like growth factor 1 (*IGF1*) Intron 2 neighbour-joining tree based on 6331 bp of phased sequence**. Branch support (>50%) is based on 1000 bootstrap replications and shown as a percentage. Dashed lines indicate the location the small dog haplotype 5 was placed in the three constraint trees. Dog breeds are italicized while grey wolf populations are normal font and listed by geographic location.

A neighbour-joining tree was constructed from the sequence data, which revealed that the common small haplotype clustered with haplotypes from Israeli grey wolves (68% bootstrap support; Figure 4). No other grey wolf population or domestic dog breed was included in this cluster. Furthermore, the small dog and all Israeli grey wolf haplotypes were ancestral to the large domestic dog haplotypes and all other grey wolf haplotypes. Haplotypes from large domestic dogs clustered with grey wolves from Alaska, Yellowstone, Italy, Spain and China (94% bootstrap support). Based on complete DNA sequence information, these results support a close affinity between the small dog haplotype and those of Middle Eastern grey wolves.

### Short Sequence (4811 bp)

In order to verify that the tree topology was not influenced by limited sampling of wild canids, eight additional grey wolf populations were sequenced for a subset of the amplicons (4811 bp; Figure 1 and Additional File 1: Table S1). Amplicons were chosen to minimize cost but maximize the number of previously discovered markers that could be sequenced (90% of SNP variation retained; Additional File 1: Table S3). Based on 28 SNPs and two indels, 21 haplotypes and three novel SNPs were identified (Additional File 4). One was private to coyotes (44230920), one was private to Israeli grey wolves (44231203) and another was found in Alaskan, Mexican, Israeli and Russian grey wolves (44227271). Grey wolf haplotypes from Israel (haplotypes 17, 18, and 20) as well as India and Iran (haplotype 20) were closest to the common small dog haplotype 4 and differed from it by 1-3 substitutions (does not include SINE and diagnostic SNP; Additional Files 3 and 4). A minimum spanning network constructed from pairwise nucleotide differences also exhibited a close relationship between the small dog haplotype (Hap 3) and haplotypes from Israel, Indian and Iranian wolves (Hap 17, 18, and 20; Figure S3). Only two haplotypes (Hap5 and Hap7) were shared between grey wolves and domestic dogs. Several grey wolf populations shared haplotype 5 with the Great Dane. These populations included Alaska, Belarus, Bulgaria, China, India, Iran, Israel, Italy and Ukraine. Dachshunds, Shih Tzu and mastiff breeds shared haplotype 7 with Alaskan, Mexican, Yellowstone, Chinese and Russian grey wolves. Nucleotide diversity was highest in coyote (0.00111, SD ± 0.0081) followed by grey wolves from the Middle East (0.00096, SD ± 0.00055; Table 2). The Middle East sample set included Israel, Iran and India. However, because Israeli grey wolf samples dominated the sample set (eight out of 11), we also estimated nucleotide diversity for each population (Table 2). Iranian grey wolf nucleotide diversity was 0.00166 (SD ± 0.00176) and Indian grey wolf was 0.00083 (SD ± 0.00062). Israeli grey wolf nucleotide diversity was 0.00101 (SD ± 0.00058). Additionally, in order to account for sampling bias in the Israeli grey wolves, two individuals were sampled 1000 times and the median nucleotide diversity was estimated to be 0.00055 (median; SD ± 0.00044). In all cases, wolves of Middle Eastern origin had the highest nucleotide diversity, second only to coyote.

**Figure 5:**
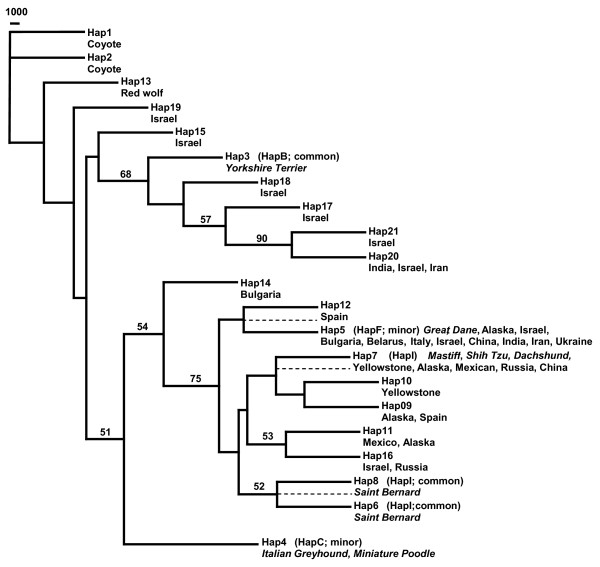
**Insulin-like growth factor 1 (*IGF1*) Intron 2 neighbour-joining tree from 4811 bp of phased sequence**. Branch support (>50%) is based on 1000 bootstrap replications and shown as a percentage. Dashed lines indicate the location the small dog haplotype 3 was placed in the three constraint trees. Dog breeds are italicized while grey wolf populations are normal font and listed by geographic location.

A neighbour-joining tree showed that the common small dog haplotype clustered with Israeli grey wolf haplotypes as well as India and Iran. This cluster was distinct from the haplotypes associated with large dogs (68% bootstrap support; Figure 5). The large dog-associated haplotypes were found to cluster with grey wolves from all populations except Bulgaria, which fell just outside of this cluster. We also observed five Israeli, one Indian and one Iranian grey wolf to be heterozygous for the large dog haplotype 5 and one of several haplotype variants (15, 17, 18, 20, 21) that clustered with the common small dog-associated haplotype (Figure 5 and Additional File 3). Again, we observe a close kinship between the small dog-associated haplotype and haplotypes observed in the Middle Eastern grey wolves.

### Constraint trees

In order to further verify the topology of the phylogenetic trees that placed the common small dog haplotype with Israeli grey wolf haplotypes, constraint trees were constructed for the long and short sequence data. In these trees, the common small dog-associated haplotype was constrained to cluster with each of the large dog haplotypes (Figures 4 and 5). For the 6331 bp dataset, three constraint trees were constructed in which the small dog-associated common haplotype (Hap 5) was placed as a sister to Hap 6, to (Hap 7, Hap 12) and to (Hap 4, Hap 8). For the 4811 bp dataset, three constraint trees were also constructed, in which Hap 3 was constrained to be sister to (Hap 5, Hap 21), to Hap 7 and to (Hap 8, Hap 6). Maximum likelihood analyses of the constraint trees confirm that the likelihoods of the unconstrained trees were significantly better in all comparisons (*P *value < 0.001).

### Recombination

Previously, a recombination point just 5' of the diagnostic SNP locus (between 44228468 and 44235098) was identified as a critical recombination locus between the common and minor small dog-associated haplotypes (haplotype B and C; see Methods) [16]. Ancestral recombination graphs from the present study suggest a similar recombination point between CanFam1 position 44230920 and 44231095 (SNPs 12, 20 in Figure S4 and 14, 15 in Figure S5). We note that 3' of this recombination point the two small haplotypes have identical sequences, whereas the 5' side exhibits several SNP differences. In order to explore the effect recombination may have had on tree topology, we constructed neighbour-joining trees for sequences on each side of the recombination point for both sequence datasets (6331 bps and 4811 bps). The topology was similar across data sets from each side of the recombination point. On the 3' side, which contains the diagnostic small dog SNP locus and SINE element, the large dog haplotypes clustered with haplotypes from all grey wolf populations, except red wolves with bootstrap support of 65% (6331 bp dataset; Figure S6) and 64% (4811 bp dataset; Figure S7). Israeli haplotypes were ancestral, or sister to, small dog haplotypes in the 6331 bp dataset (Figure S6) and small dog haplotypes were ancestral to those in all dogs in the 4811 bp dataset (Figure S7). On the 5' side of the recombination point, many of the grey wolf haplotypes and large domestic dog haplotypes were identical. All but the common small dog haplotype, and several of the Israeli (as well as Indian, Iranian and Russian) grey wolf haplotypes, clustered with support values of 97% (6331 bp dataset; Figure S8) and 88% (4811 bp dataset; Figure S9).

## Discussion

Previous research identified *IGF1 *as a major gene affecting skeletal size in domestic dogs [16]. In this study, we examined genetic variation surrounding the *IGF1 *gene in the progenitor of domestic dogs in order to uncover the evolutionary history of the gene. This study confirms the absence of the derived small SNP allele in the intron 2 region of *IGF1 *(CanFam1 44228468) in a large sample of grey wolves and further establishes the absence of a small dog associated SINE element in all wild canids and most large dog breeds. Thus, the absence of both the SINE element and SNP allele in wild canids suggests that the mutation for small body size post-dates the domestication of dogs. Presumably, the absence of these two loci in wolves may reflect a unique recombination event in domestic dogs. However, we find no evidence of recombination between the SINE element and derived SNP allele in domestic dogs and the derived SNP allele distinguishes the associated common small (A, B and C) and large (D-L) haplotypes. Additionally, because all small dogs possess these diagnostic mutations, the small size phenotype likely arose early in the history of domestic dogs.

Although the alleles distinguishing small domestic dogs from large domestic dogs have been identified, the causal mutation for small body size has not been definitively determined. Microsatellite mutations have been suggested as a potential source for rapid morphological evolution in domestic dogs and the microsatellite located in the promoter region of *IGF1 *could conceivably be the causal mutation [18]. However, we found that the allelic range observed in large and small domestic dogs was similar to that in grey wolves and coyotes, which does not support this hypothesis. Synonymous mutations in coding regions have been proposed to regulate gene expression and splicing [19-21]. From analysis of sequencing data, we found that the synonymous SNP mutation within exon 3 was segregating in both large and small domestic dogs as well as grey wolves, which does not support this hypothesis. Although additional sequencing is needed in order to identify other possible variants, the unique derived SINE element and the SNP allele in intron 2 cannot be ruled out as candidates for the causative mutation. SINE elements have been found to be widely distributed throughout the dog genome and segregating in some breeds while fixed in others [2,22]. Previous studies in domestic dogs have identified SINE elements affecting coat colour variation, hearing and sight disorders, narcolepsy, and myopathy [23-25]. Additionally, Alu elements, the most frequent SINE retrotransposon in humans, have been shown to affect gene regulation and splicing of mRNA [26-28]. However, in the absence of any functional studies, any conclusions about the role of the SNP allele and the SINE element in size variation are speculative. Furthermore, the SNP allele and SINE element are not known to be associated with any regulatory elements or splicing site.

We investigated the progenitor population for the small body size mutation by examining the PCA of 94 dog-derived SNP genotypes in a range of potential ancestral grey wolf populations. The patterns observed on the first two axes of variation were consistent with known evolutionary history. On the first axis of variation, we observed separation of domestic dogs and grey wolves, whereas along the second axis of variation we observed a large separation between large and small domestic dogs and a smaller separation between Old World and New World wolves. We also observed a slightly closer kinship of Middle Eastern grey wolves with domestic dogs. Thus, SNP genotype data reveal ancient and recent evolutionary relationships in wild and domestic canids.

In order to explore this relationship further, we analysed sequence data directly spanning the diagnostic SINE element and SNP locus. Nucleotide diversity estimates in grey wolf populations were highest in Middle Eastern grey wolves for the 4811 bp dataset and European grey wolves for the 6331 bp dataset. However, the 4811 bp dataset contained the greatest number of samples per group, which should more accurately represent diversity estimates. In this dataset, Middle Eastern grey wolves had the greatest nucleotide diversity even when sampling variation was taken into account by equalizing the number of samples in the population. Haplotypes from Middle Eastern grey wolves were consistently found to have the greatest similarity to those in small domestic dogs for both the 6331 bp and 4811 bp datasets. Additionally, phylogenetic analyses suggested a closer kinship of the common small domestic dog haplotype with Middle Eastern grey wolf haplotypes. Although bootstrap support values are not high, SNP PCA and sequence analysis all are concordant with a Middle Eastern origin for the small domestic dog haplotypes.

## Conclusions

The Middle East includes part of the Fertile Crescent where farming began and was the origin of many domesticated plants and animals including cereals, cats and goats [29-32]. The region has been suggested as the site of dog domestication based on archeological data [33,34]. Specifically, several archaeological sites in the Middle East have some of the earliest domestic dog remains, dating to 12,000 years ago [15,33]. However, sites in Belgium, Germany and Western Russia contain older remains (13,000-31,000 years ago) [12,13]. Many of the domestic dogs from Middle Eastern sites are small whereas those from Belgium, Germany and Western Russia are larger in size, which supports our hypothesis that small body size evolved early in the history of domestic dogs and probably in the Middle East. Reduction in body size is a common feature of domestication and appears early in other domesticated taxa including cattle, pigs and goats [35-37]. Indeed it is a morphological attribute researchers have used to distinguish early domestic dogs from their grey wolf progenitor [33,34]. Other features include shortening of the muzzle, large crowned teeth and paedomorphic characteristics. Therefore, in concordance with past archeological studies, our molecular analysis provides strong evidence for the early evolution of small size in dogs in the Middle East, more than 12,000 years ago.

Genetic studies exploring the evolutionary history of domestic dogs have focused primarily on mtDNA [4-6,11,38,39]. Phylogenetic analysis and haplotype diversity of mtDNA sequence data from a global sampling of grey wolves and domestic dogs specifically suggested an East Asian origin for domestic dogs [4,11]. By contrast, our results show that the small dog haplotype is closely related to haplotypes in wolves from the Middle East and is consistent with an ancient origin in this region of small domestic dogs. Small dogs have been recorded in 10,000 to 12,000 year old burial sites in the Levant [15,33] and new SNP data further suggest it as a primary centre for dog domestication [40]. The lack of concordance between mtDNA and nuclear analysis could reflect differences in sampling, a female/male bias in dispersal or breed specific bias in inbreeding and population size [4,41-43]. As well as providing information about population history, genes controlling morphological traits can provide direct information about the selective and cultural context of domestication. Small size could have been more desirable in more densely packed agrarian societies where dogs may have lived partly indoors or in confined outdoor spaces. This study provides further evidence for the importance of a major size gene early in the evolutionary history of dogs and implicates the Levant culture as an initial source and selective agent for small size in domestic dogs.

## Methods

### Datasets

Four molecular datasets were utilized in this study: (1) SNP genotypes; (2) microsatellite genotypes; (3) SINE element genotypes; and (4) DNA sequences. The datasets were generated at different times and contained different but overlapping sets of samples (Additional File 1: Table S1). First, for a broad exploration of the *IGF1 *gene region, 94 SNP loci were genotyped utilizing the SNPlex system (Applied Biosystems, CA, USA). These SNPs are a subset of 116 SNPs that successfully amplified in domestic dogs and wild canids and were originally characterized in Sutter *et al*. [16]. They were ascertained in nine dogs from small and large breeds that were chosen to cover all major *IGF1 *haplotypes identified to that point. The SNPs span the entire *IGF1 *gene (Figure 1; Additional File 1: Table S2). Samples genotyped include 15 large domestic dog breeds (>30 kg; *n *= 234), 23 small domestic dog breeds (<9 kg; *n *= 340), 11 grey wolf populations (*n *= 119) and one coyote population (*n *= 21) (Additional File 1: Table S2).

Second, a dinucleotide microsatellite (CA_n_; CanFam1 44283699-44283736) in the promoter region of *IGF1*, that was previously found to have a significant association to body size [16], was typed in 18 small domestic dog breeds (*n = 554*), 13 large domestic dog breeds (*n = 390*), 16 grey wolf populations (*n = 388*) and two coyote populations (*n = 54*).

Third, the antisense-oriented retrotransposon (SINEC_Cf; CanFam1 44228010-44228230) and derived SNP allele (CanFam1 44228468), which are diagnostic for small dogs, were genotyped in 17 grey wolf populations (*n = 374*) and six distantly related species including the coyote (*n = 100*), golden jackal (*n = 16*), Ethiopian wolf (*n = 20*), bat-eared fox (*n = 20*), grey fox (*n = 33*) and Channel Island fox (*n = 26*).

Finally, DNA sequencing of *IGF1 *was carried out and performed in two stages (Additional File 1: Table S1 and S2). First, 6331 bp directly surrounding the SNP and SINE element of the intron 2 region of *IGF1 *were sequenced using Sanger sequencing. Samples sequenced included: six grey wolf populations (*n = 10*) and seven domestic dog breeds (*n = 8*). In small dogs, one common haplotype (B) and one minor haplotype (C) were previously found to be diagnostic for small body size (see Figure 3b and 3c of Sutter *et al*. [16]). Likewise, in large dogs, one common haplotype (I) and one minor haplotype (F) were diagnostic for large body size. Five domestic dogs were selected that were homozygous for each of the large dog haplotypes and three domestic dogs were selected that were homozygous for the small dog haplotypes. The shih tzu samples were selected because they were homozygous for the common large dog haplotype, which was previously observed in several small dogs [16]. Second, an additional eight grey wolf populations (*n = 10*) and two Dachshunds were sequenced for 4811 bp across the same region. We increased our sampling to ensure that we captured all potential source populations for small dogs. However, in order to reduce effort and cost, we sequenced only amplicons which were found to contain the majority of the SNP variation (~90%; Additional File 1: Table S3).

Blood, tissue or buccal swabs were collected from sampled individuals. Genomic DNA from blood and tissue was extracted by a standard phenol-chloroform protocol. DNA from buccal swabs was extracted using the Blood Midi Kit (Qiagen, CA, USA). All samples were stored at -20°C for short-term storage and -80°C for long-term storage. For samples with low DNA concentrations, whole genome amplification was performed using the REPLI-g kit according to manufacturer guidelines (Qiagen). All domestic dog sampled were purebred and registered with the American Kennel Club. Pedigrees were used to choose samples that were unrelated to one another at the grandparent level. Grey wolf samples were chosen to be globally distributed and representative of all major populations. SNP genotypes were amplified on the SNPlex system following the manufacturer's guidelines (Applied Biosystems, CA, USA). Bi-directional sequences were polymerase chain reaction amplified and cleaned with exonuclease/shrimp alkaline phosphate following standard protocols. Primers for all loci and sequenced amplicons can be found in the supplemental material of Sutter *et al*. [16] (see Additional File 1: Tables S2 and S3 for SNP and sequence position). Genotype and sequence data were collected on an ABI 3730 (Applied Biosystems). Genemapper 4.0 was used to make genotype calls for each SNP, microsatellite, and SINE element locus (Applied Biosystems). Sequence polymorphisms were identified and viewed using Phred/Phrap/Consed/Polyphred [44-47].

### Analysis

PCA was performed using Egigenstrat [48]. The software program PHASE was used to infer haplotypes across each species and region (that is, domestic dogs, New World grey wolves, Old World grey wolves, coyotes) [49,50]. Phased haplotypes were used as the operational taxonomic unit in phylogenetic analysis (see below). Lastly, Arlequin was used to calculate nucleotide diversity and minimum spanning networks [51].

Prior to phylogenetic analysis, jModelTest [52] was run on the sequence data to determine the best mutation model, transition/transversion ratio (ti/tv) and gamma distribution parameter. Phylogenetic analysis was performed on phased haplotypes using PHYLIP [53]. Majority-rule consensus neighbour-joining trees were constructed from 1000-10,000 bootstrap replications. Constraint trees were generated with the Retree function and then run under maximum likelihood in PHYLIP in order to compare resulting likelihood values to the unconstrained tree. For the full 6331 bp sequence dataset, an F81 mutation model was used with a gamma distribution parameter of 0.015 and ti/tv ratio of 4.6. For the partial 4811 bps sequence dataset, an F81 mutation model was used with a gamma distribution parameter of 0.0110 and a ti/tv ratio of 6.8. The software SHRUB was used to construct ancestral recombination graphs http://www.cs.ucdavis.edu/~yssong/lu.html. SHRUB uses a branch and bound method to calculate the minimum number of recombination events necessary to explain the data.

## Abbreviations

Bp: base pair; *IGF1*: insulin-like growth factor 1; LD: linkage disequilibrium; PCA: principal components analysis; SD: standard deviation; SNP: single nucleotide polymorphism; ti/tv: transition/transversion ratio.

## Authors' contributions

All authors contributed to the conception, design and coordination of the study. MMG and NBS carried out the laboratory work. MMG analysed the data, interpreted the results and drafted the manuscript. All authors read, edited and approved the final manuscript.

## Supplementary Material

Additional file 1 **Supplemental Material**. This PDF file contains the following: Figure S1: Neighbour-joining tree from insulin-like growth factor 1 (*IGF1*) dog derived genotyped single nucleotide polymorphisms (SNPs). Figure S2: Minimum spanning network of 6331 bps of phased sequence. Figure S3: Minimum spanning network of 4881 bps of phased sequence. Figure S4: Ancestral recombination graph of 6331 bps of phased sequence. Figure S5: Ancestral recombination graph of 4811 bps of phased sequence. Figure S6: Neighbour-joining tree based on 6331 bps of phased sequences from the 3' side of the recombination point. Figure S7: Neighbour-joining tree based on 4811 bps of phased sequences from the 3' side of the recombination point. Figure S8: Neighbour-joining tree based on sequences from the 5' side of the recombination point totaling 6331 bp. Figure S9: Neighbour-joining tree based on sequences from the 5' side of the recombination point totaling 4811 bp. Table S1: Sample datasets for domestic and wild canids used in each of five marker assays. Table S2: Dog-derived SNPs and sequence discovered SNPs and indels. Table S3: Sequenced amplicons across intron 2 of *IGF1*. Click here for file

Additional file 2 **Dog-derived single nucleotide polymorphism (SNP) marker haplotypes based on 20 SNPs **[15]. Counts are number of chromosomes. Greyed cells are the derived allele determined from golden jackal sequences. Bold haplotypes are small dog haplotypes. Italicized haplotypes are those with the least number of differences from small dog haplotype B. Click here for file

Additional file 3 **Single nucleotide polymorphism haplotypes from sequenced regions of intron 2 **(Figure 1). Counts are number of chromosomes. Grey vertical bar indicates a recombination point. Greyed cells are the derived allele determine from ancestral golden jackal sequences. Bold haplotypes are small dog haplotypes. Click here for file

Additional file 4 **Matrix of single nucleotide polymorphism differences between haplotypes from 4811 bp of sequence**. Bold haplotypes indicate small dog haplotypes. Bold numbers indicate haplotypes with smallest number of differences to the common small dog haplotype B. Click here for file
