# The *IGF1 *small dog haplotype is derived from Middle Eastern gray wolves

**DOI:** 10.1186/1741-7007-8-118

**Published:** 2010-09-08

**Authors:** Melissa M Gray, Nathan B Sutter, Elaine A Ostrander, Robert K Wayne

**Affiliations:** 1 Department of Ecology and Evolutionary Biology, University of California, Los Angeles, CA, USA; 2 Laboratory of Genetics, University of Wisconsin, Madison, WI, USA; 3 Department of Clinical Sciences, College of Veterinary Medicine, Cornell University, Ithaca, NY, USA; 4 Cancer Genetics Branch, National Human Genome Research Institute, National Institutes of Health, Bethesda, MD, USA

## Correction

The authors note minor corrections to two figures which originally appeared in Gray et al. in 2010 [1]. In Figure S1, a Chinese grey wolf terminus was mislabeled as an Old world grey wolf. In Figure S3, there should be two mutational steps between Hap14 and Hap05 instead of one. The corrected figures are presented below (Fig 1 (Figure S1 in [1]) & Fig 2 (Figure S3 in [1])). These changes do not affect any of the conclusions presented in the original manuscript.

**Figure 1:**
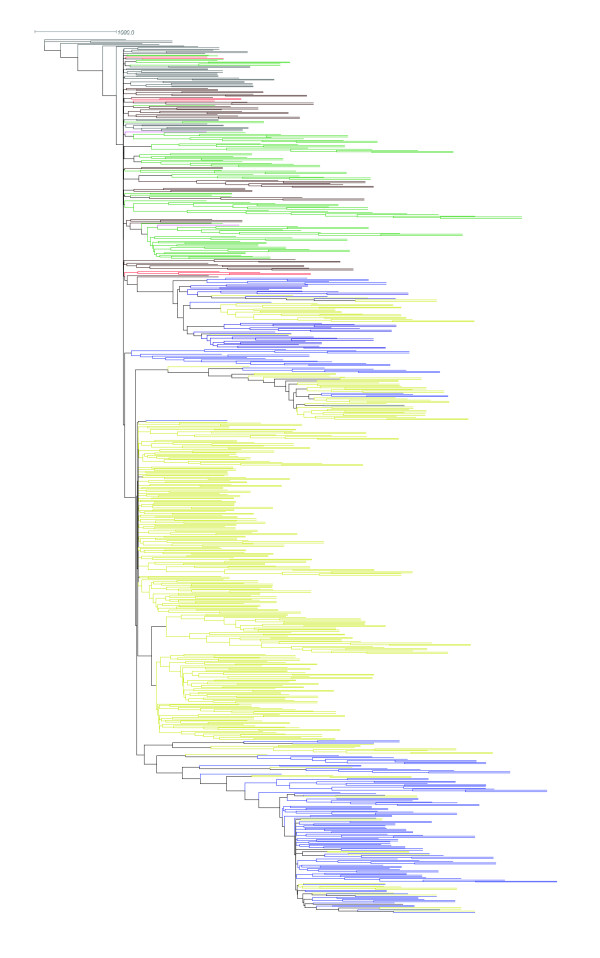
**Neighbor-joining tree from *IGF1 *dog derived genotyped SNPs**. The following populations are included: coyotes (grey), New World wolves (green), Old World wolves (brown), Middle East wolves (red), Chinese wolves (pink), small domestic dogs (yellow), giant domestic dogs (blue). A 1000 bootstrap majority-rule consensus tree constructed on phased haplotypes under a Kimura-2paramter mutation model with a gamma distribution of 0.946 and a ti/tv ratio of 3.85 is shown.

**Figure 2:**
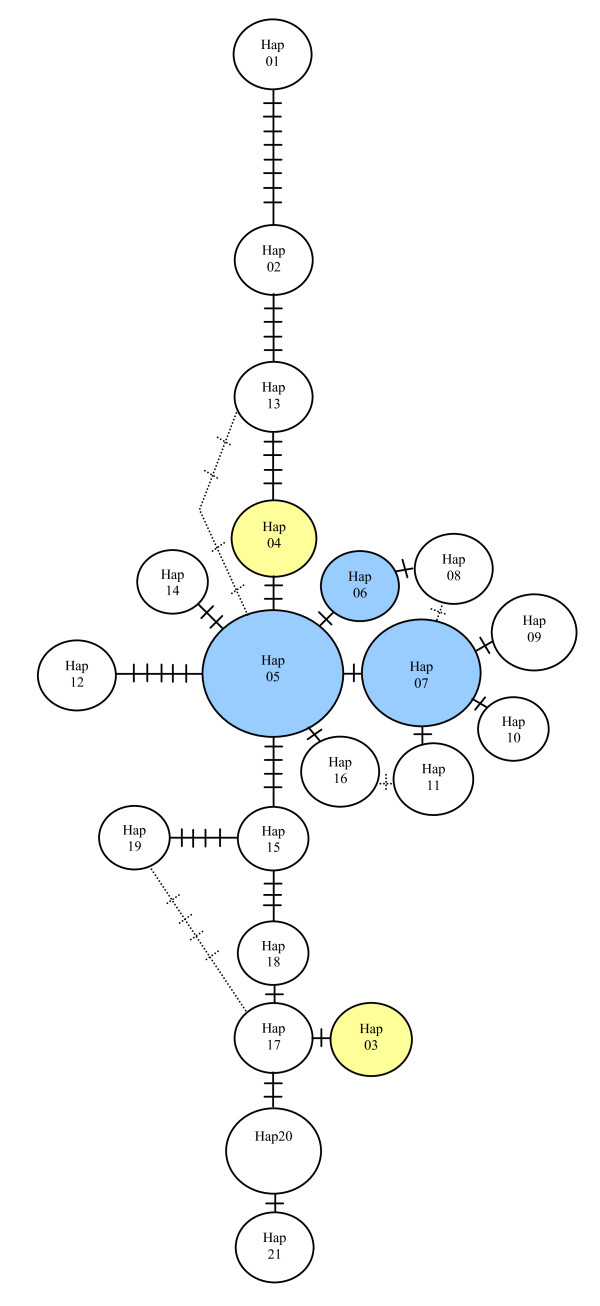
**Minimum spanning network of 4881 bps of phased sequence**. See Figure 5 for specific breed and grey wolf haplotype labels. Node size correlates to the frequency of the haplotype across all samples. Hashes indicate the number of pairwise differences between haplotypes. Yellow nodes indicate the small dog *IGF1 *haplotypes and the blue nodes indicate the large dog *IGF1 *haplotypes. Dashed lines display alternative connections between haplotypes.

We apologize for any inconvenience caused by this error.
