# Supplementary material for: The IGF1 small dog haplotype is derived from Middle Eastern grey wolves
Source: BMC Biol. 2010 Feb 24;8:16. doi: 10.1186/1741-7007-8-16 (PMC2837629; doi:10.1186/1741-7007-8-16)
Supplement: Additional file 1 — Supplemental Material. This PDF file contains the following: Figure S1: Neighbour-joining tree from insulin-like growth factor 1 (IGF1) dog derived genotyped single nucleotide polymorphisms (SNPs). Figure S2: Minimum spanning network of 6331 bps of phased sequence. Figure S3: Minimum spanning network of 4881 bps of phased sequence. Figure S4: Ancestral recombination graph of 6331 bps of phased sequence. Figure S5: Ancestral recombination graph of 4811 bps of phased sequence. Figure S6: Neighbour-joining tree based on 6331 bps of phased sequences from the 3' side of the recombination point. Figure S7: Neighbour-joining tree based on 4811 bps of phased sequences from the 3' side of the recombination point. Figure S8: Neighbour-joining tree based on sequences from the 5' side of the recombination point totaling 6331 bp. Figure S9: Neighbour-joining tree based on sequences from the 5' side of the recombination point totaling 4811 bp. Table S1: Sample datasets for domestic and wild canids used in each of five marker assays. Table S2: Dog-derived SNPs and sequence discovered SNPs and indels. Table S3: Sequenced amplicons across intron 2 of IGF1. [file 1741-7007-8-16-S1.PDF]

Supplemental Material

Figures

Figure S1 - Neighbor-joining tree from *IGF1* dog derived genotyped SNPs.

The following populations are included: coyote (grey), New World wolves (green), Old World wolves (brown), Middle East wolves (red), Chinese wolves (pink), small domestic dogs (yellow), giant domestic dogs (blue). A 1000 bootstrap majority-rule consensus tree constructed on phased haplotypes under a Kimura-2paramter mutation model with a gamma distribution of 0.946 and a ti/tv ratio of 3.85 is shown.

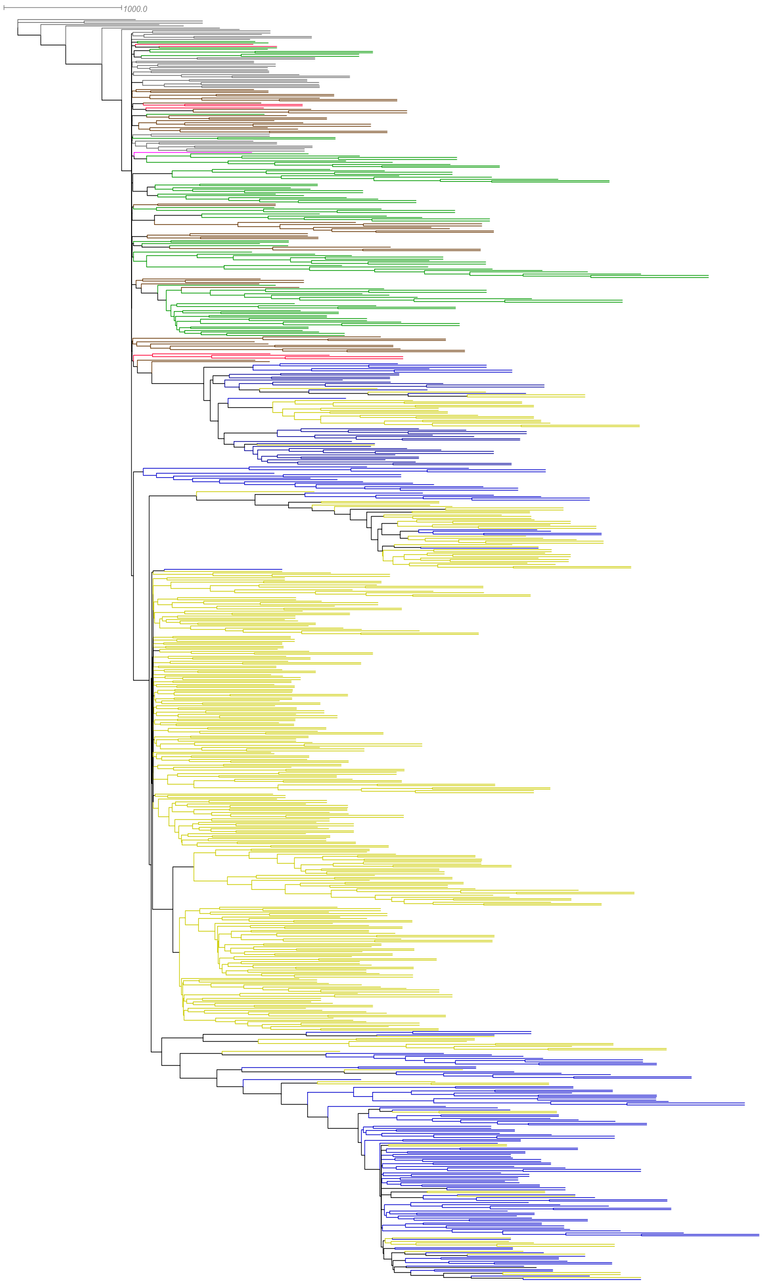

Figure S2 - Minimum spanning network of 6331 bps of phased sequence.

See Figure 4 for specific breed and gray wolf haplotype labels. Node size correlates to the frequency of the haplotype across all samples. Hashes indicate the number of pairwise differences between haplotypes. Yellow nodes indicate the small dog *IGF1* haplotypes, which the blue nodes indicate the large dog *IGF1* haplotypes.

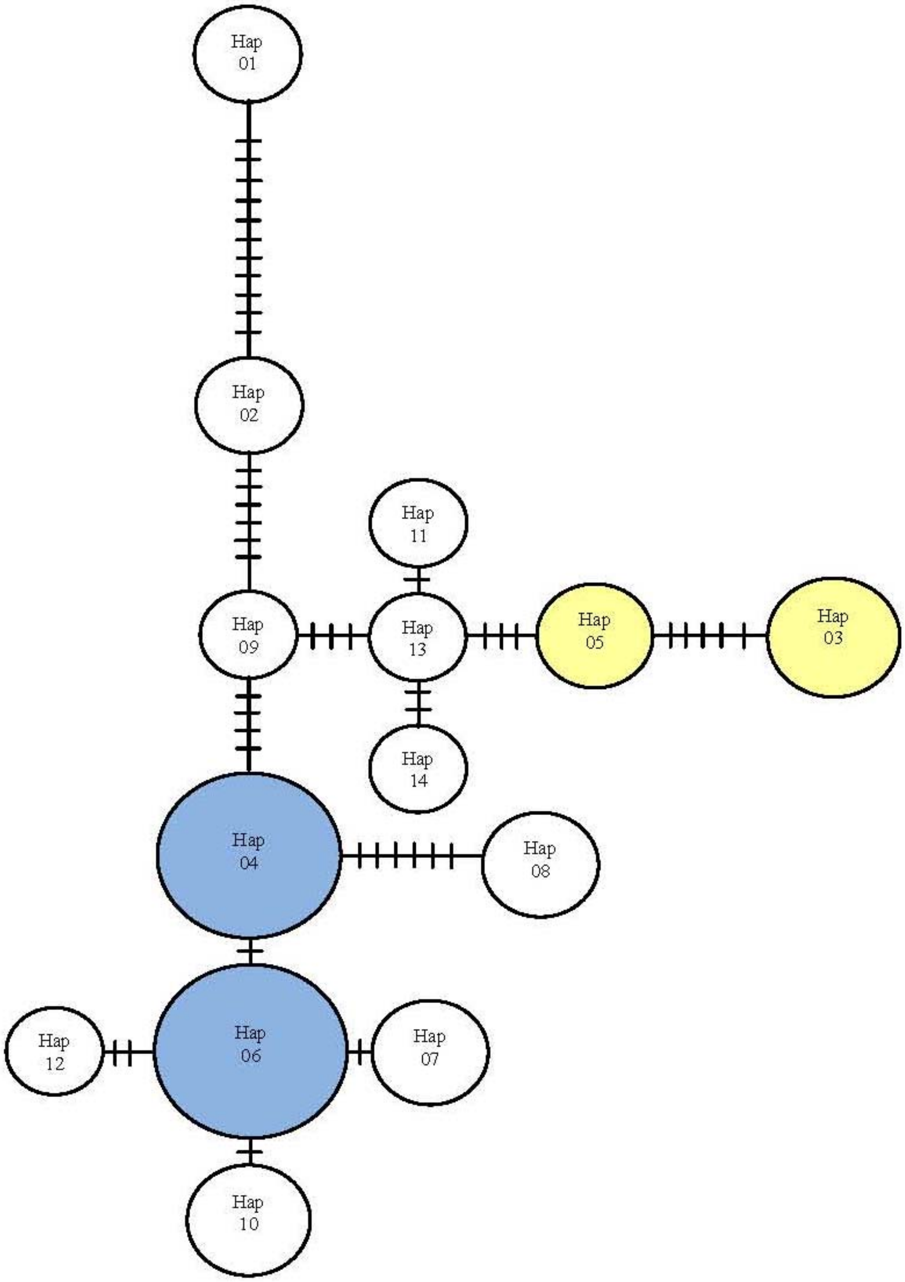

Figure S3 - Minimum spanning network of 4881 bps of phased sequence.

See Figure 5 for specific breed and gray wolf haplotype labels. Node size correlates to the frequency of the haplotype across all samples. Hashes indicate the number of pairwise differences between haplotypes. Yellow nodes indicate the small dog *IGF1* haplotypes, which the blue nodes indicate the large dog *IGF1* haplotypes. Dashed lines display alternative connections.

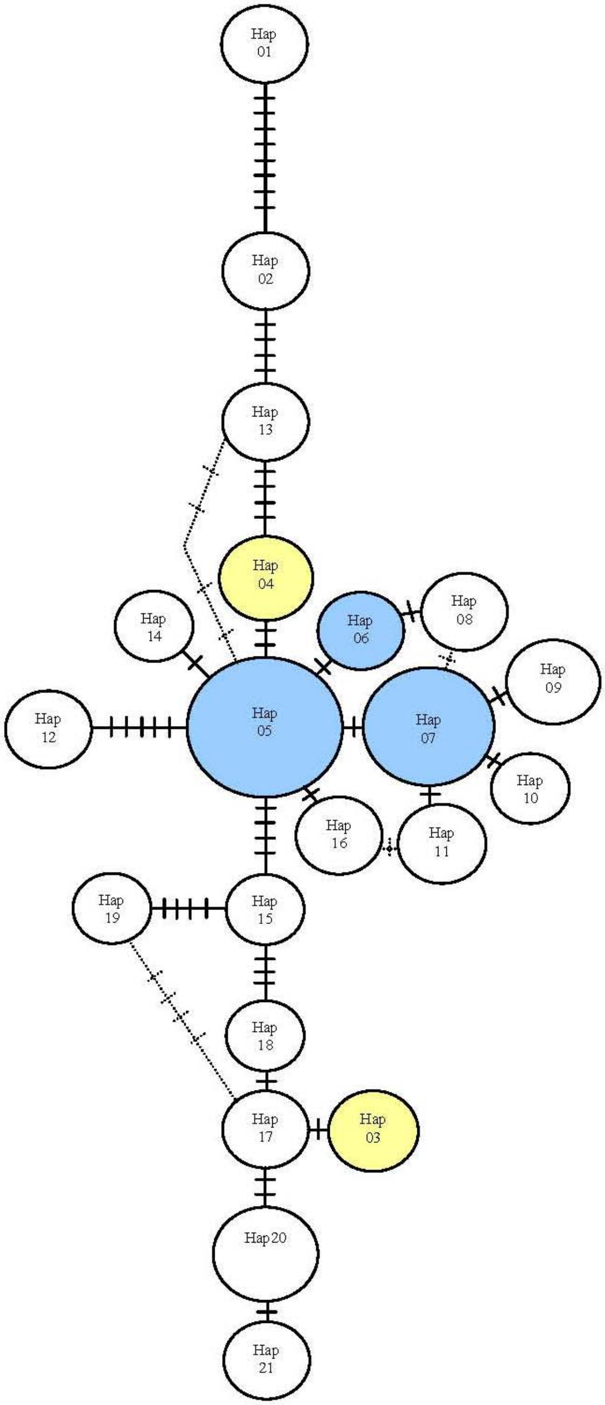

Figure S4 - Ancestral Recombination Graph of 6331 bps of phased sequence.

Red dots represent the haplotypes from Table 3, white dots represent coalescent events, and blue dots represent recombination events. The numbers in parenthesis indicate the breakpoint of recombination between the two SNP loci and the numbers correspond to SNPs from left to right as presented in Table 3. The numbers on the edges indicate mutation events. “L” and “R” indicate the left or right, respectively, of the recombination break point.

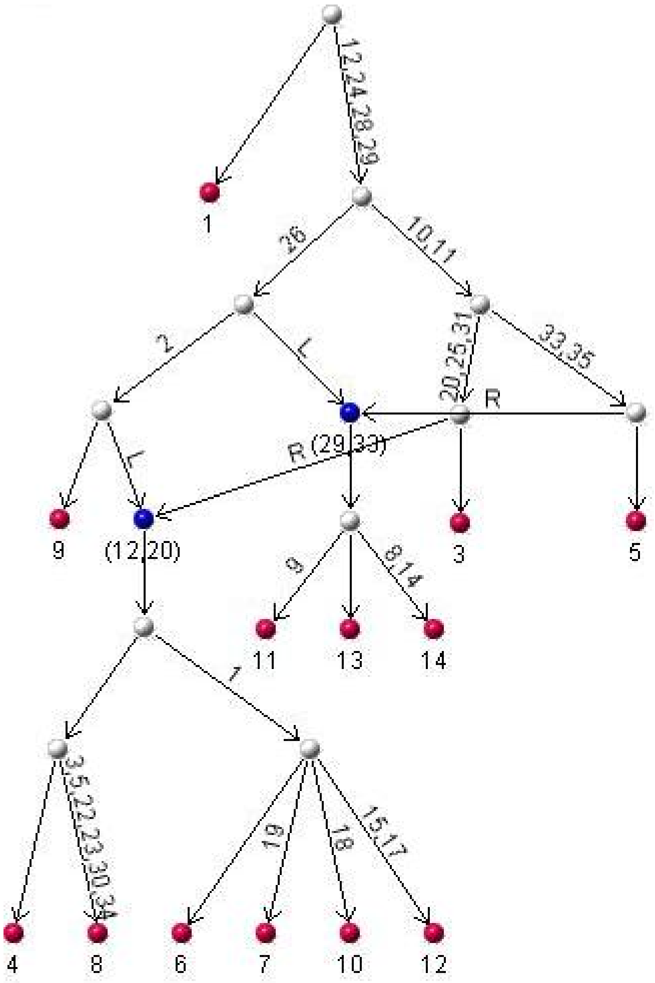

Figure S5 - Ancestral Recombination Graph of 4811 bps of phased sequence.

Red dots represent the haplotypes from Table 3, white dots represent coalescent events, and blue dots represent recombination events. The numbers in parenthesis indicate the breakpoint of recombination between the two SNP loci and the numbers correspond to SNPs from left to right as presented in Table 3. The numbers on the edges indicate mutation events. “L” and “R” indicate the left or right, respectively, of the recombination break point.

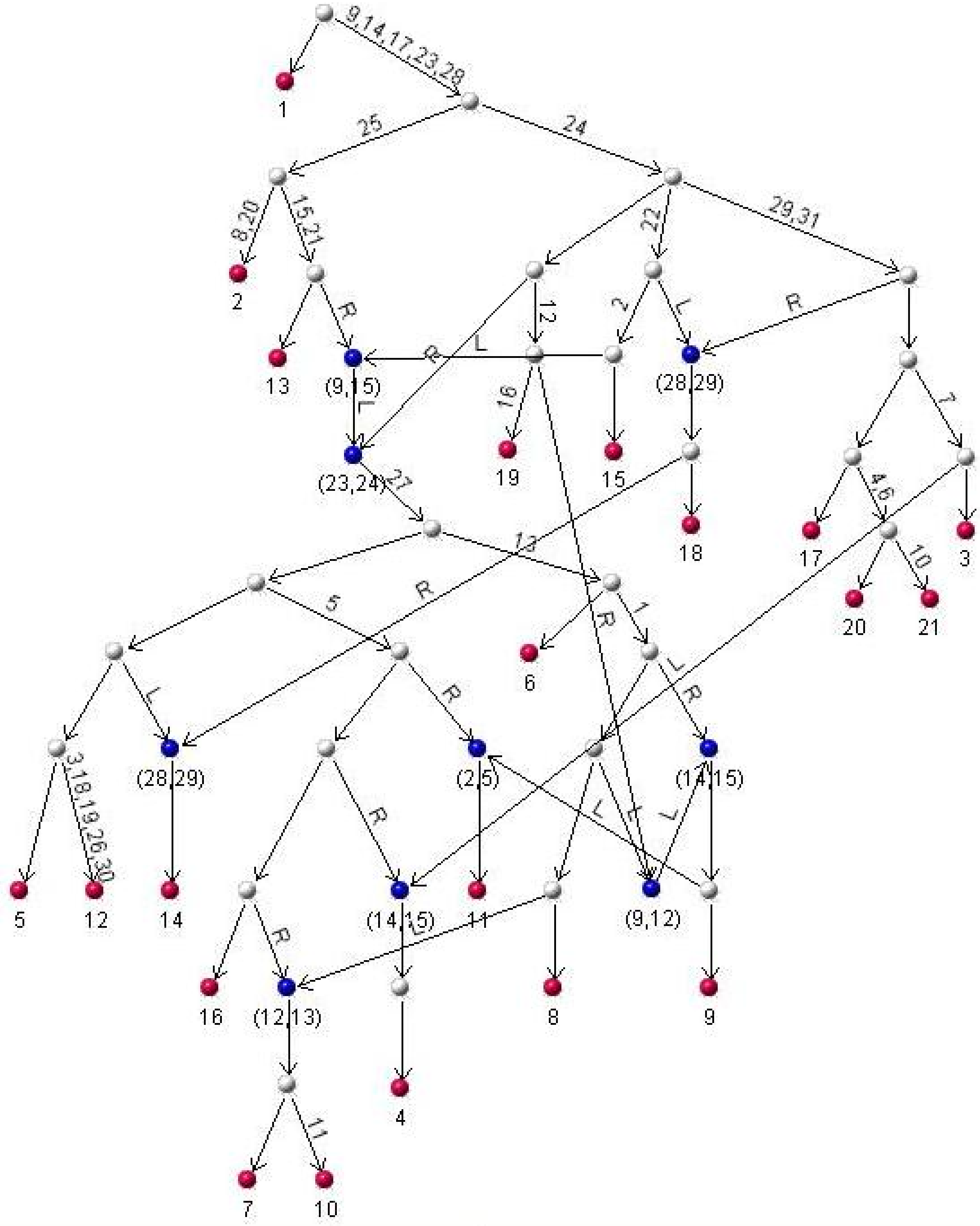

Figure S6 - Neighbor-joining tree based on 6331 bps of phased sequences from the 3' side of the recombination point. Branch support (>50%) is based on 1000 bootstrap replications and shown as a percentage. Dog breeds are italicized while gray wolf populations are normal font and listed by geographic location.

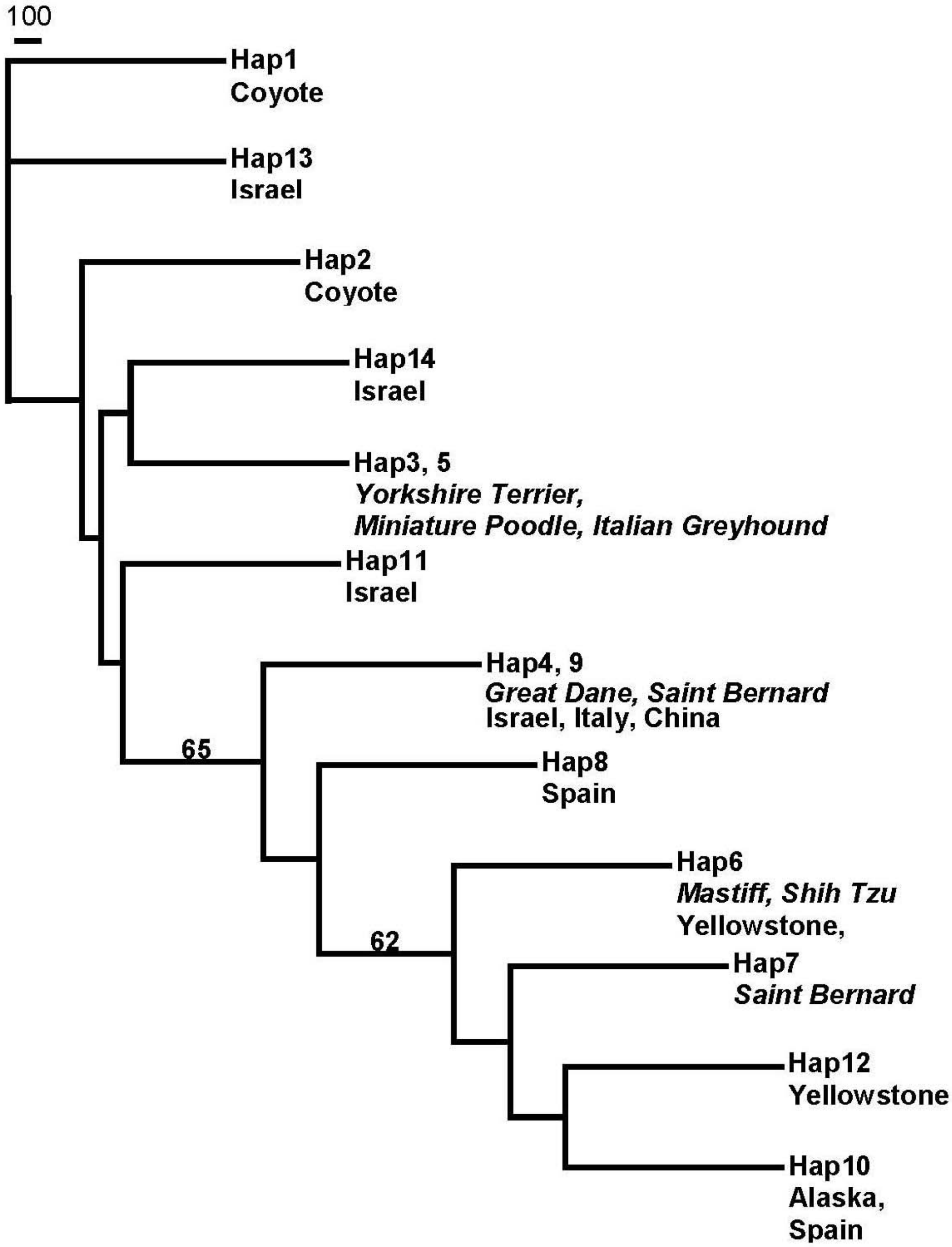

Figure S7 - Neighbor-joining tree based on 4811bps of phased sequences from the 3' side of the recombination point. Branch support (>50%) is based on 1000 bootstrap replications and shown as a percentage. Dog breeds are italicized while gray wolf populations are normal font and listed by geographic location.

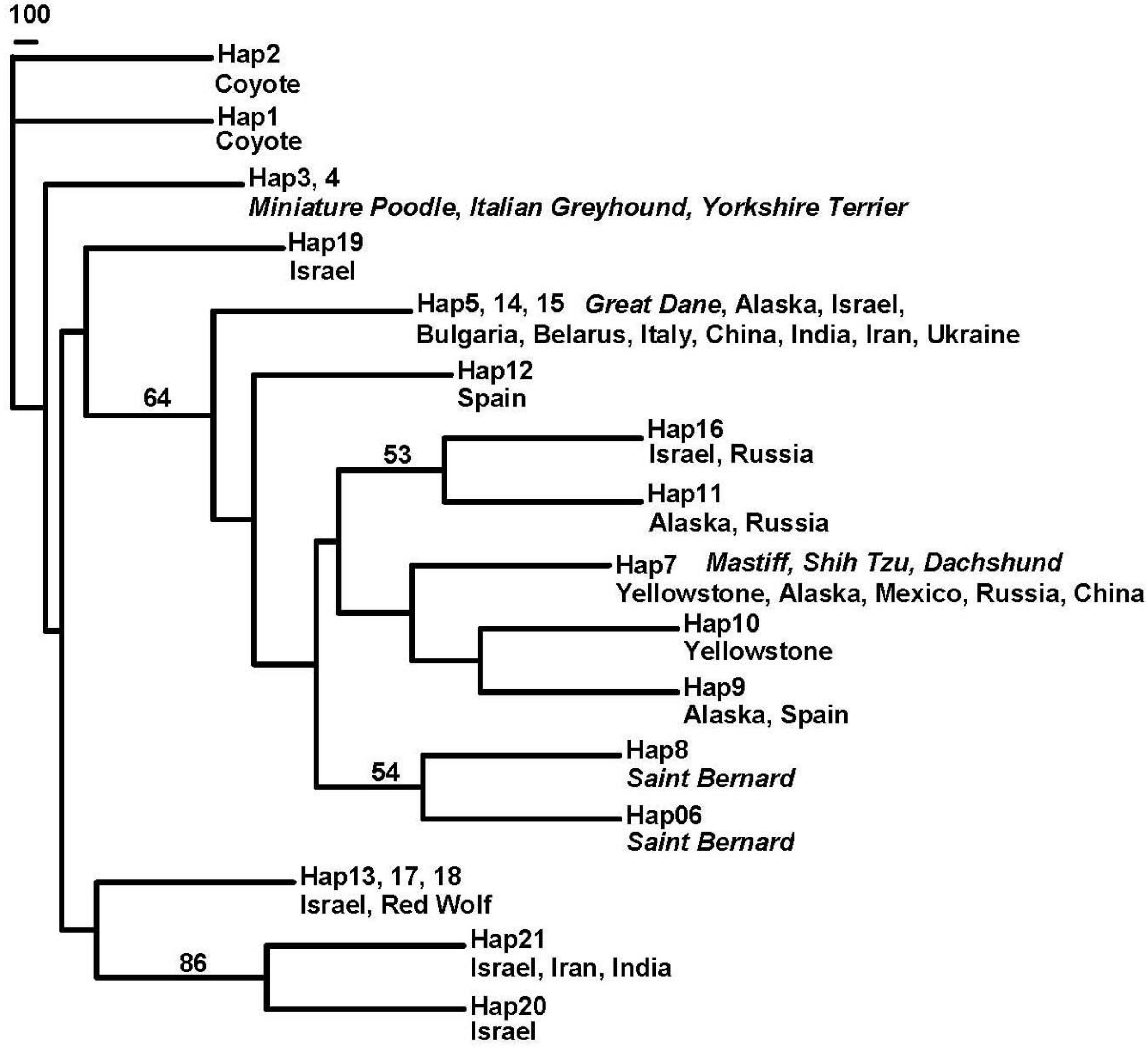

Figure S8 - Neighbor-joining tree based on sequences from the 5' side of the recombination point totaling 6331 bp. Branch support (>50%) is based on 1000 bootstrap replications and shown as a percentage. Dog breeds are italicized while gray wolf populations are normal font and listed by geographic location.

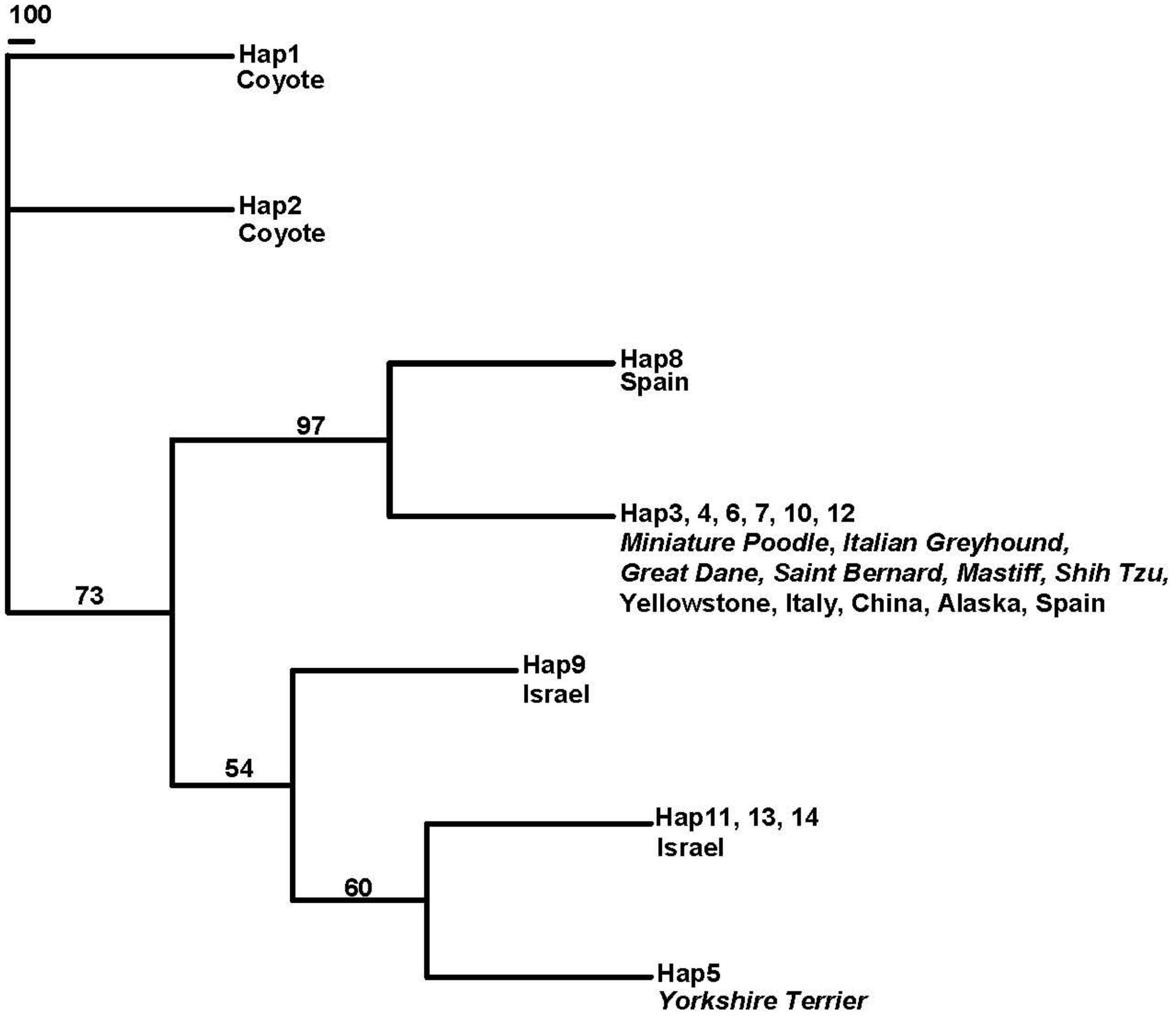

Figure S9 - Neighbor-joining tree based on sequences from the 5' side of the recombination point totaling 4811 bp. Branch support (>50%) is based on 1000 bootstrap replications and shown as a percentage. Dog breeds are italicized while gray wolf populations are normal font and listed by geographic location.

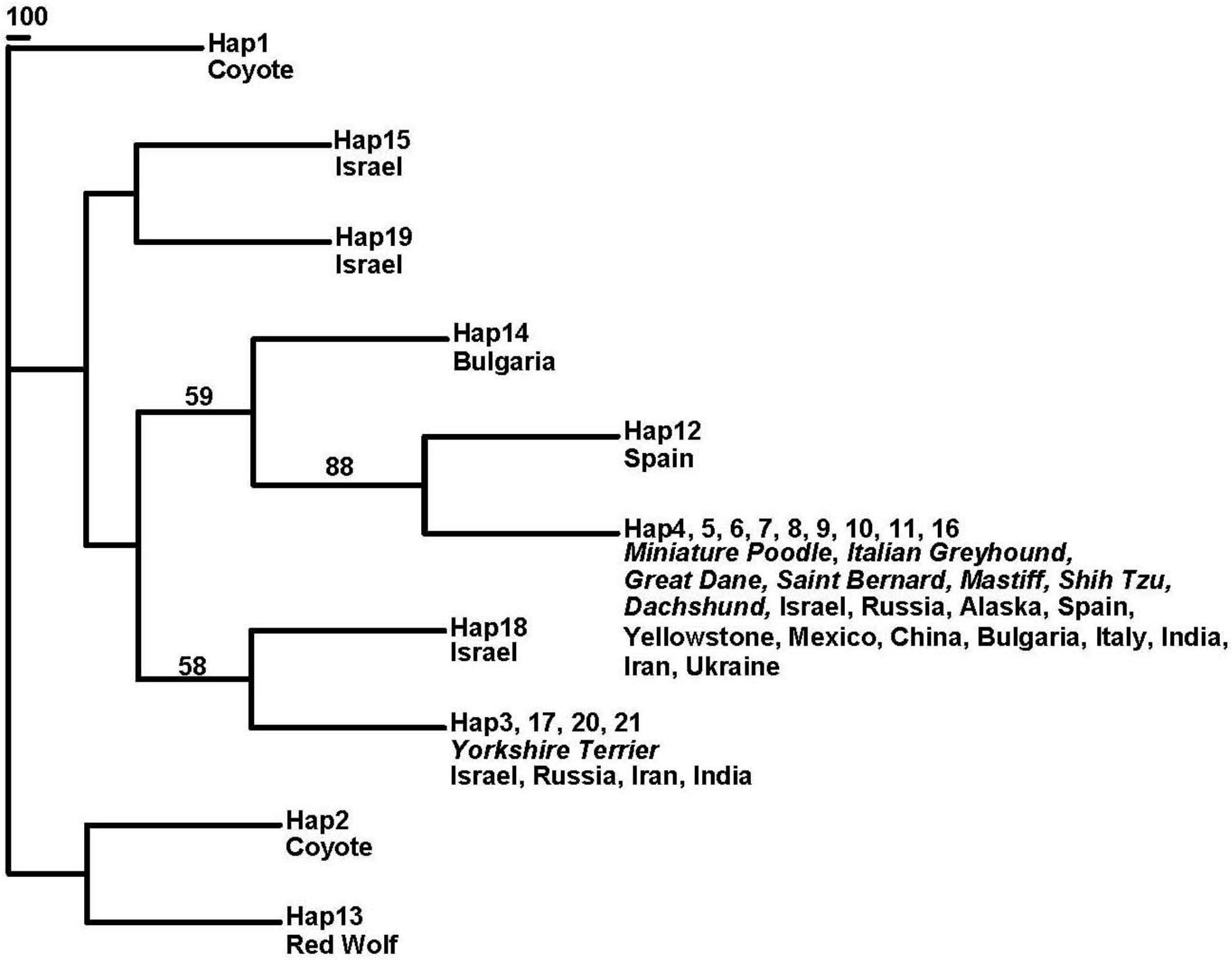

Tables

Table S1 - Sample datasets for domestic and wild canids used in each of five marker assays.

Assay		SNPs	Indels	Number of Populations: Coyote	Gray Wolf	Large Dogs (>30 kg)	Small Dogs (<9kg)	Other Canid Species
1. SNPs		94		One population (n=21)<br>California	11 populations (n=119)<br>Alaska, Banff (Canada), China, India, Isle Royale, Israel, Italy, Northern Quebec, Spain, Sweden, Yellowstone	15 breeds (n=234)<br>Akita, Alaskan malamute, Bernese mountain dog, borzoi, bull mastiff, great Dane, great Pyrenees, Irish wolfhound, kuvasz, mastiff, Newfoundland, Rottweiler, Saint Bernard, Scottish deerhound, giant schnauzer	23 breeds (n=340)<br>Chihuahua, bichon frise, border terrier, Boston terrier, Brussels griffon, Cavalier King Charles spaniel, Chinese crested, English toy spaniel, Italian greyhound, Jack Russell terrier, Japanese chin, Maltese, miniature schnauzer, Norfolk terrier, Norwich terrier, Papillion, Pekingese, Pomeranian, Pug, Shih Tzu, silky terrier, toy fox terrier, Yorkshire terrier	
2. Microsatellite				Two populations (n=54)<br>California, Washington	16 populations (n=388)<br>Alaska, Canada, China, Croatia, India, Isle Royale, Israel, Italy, Mexican, Minnesota, Northern Quebec, North West Territory, Red Wolf, Spain, Sweden, Yellowstone	13 breeds (n=390)<br>Akita, Bernese mountain dog, bullmastiff, giant schnauzer, great Dane, great Pyrenees, Irish wolfhound, mastiff, Newfoundland, presa canario, Rottweiler, Saint Bernard, Tibetan mastiff	18 breeds (n=554)<br>border terrier, Boston terrier, Cavalier King Charles spaniel, Chihuahua, Chinese crested, Italian greyhound, Jack Russell terrier, Japanese chin, miniature poodle, miniature schnauzer, Norwich terrier, Papillion, Pekingese, Pomeranian, Shih Tzu, toy fox terrier, toy poodle, Yorkshire terrier	
3. SINE element				Two populations (n=100)<br>California, Washington	17 populations (n=374)<br>Alaska, Canada, China, Croatia, India, Isle Royale, Israel, Italy, Mexico, Minnesota, Northern Quebec, North West Territory, Oman, Red Wolf, Spain, Sweden, Yellowstone			5 populations (n=115)<br>Golden Jackal, Ethiopian Wolf, Bat-eared Fox, Gray Fox, Channel Island Fox
4. Sequence	6331bp	30	4	One populations (n=2)<br>California	6 populations (n=10)<br>Alaska, China, Israel, Italy, Spain, Yellowstone	4 breeds (n=5)<br>great Dane, mastiff, Saint Bernard, Shih Tzu	3 breeds (n=3)<br>Italian greyhound, miniature poodle, Yorkshire terrier	
5. Sequence	4811bp	28	2	0ne population (n=2)<br>California	14 populations (n=28)<br>Alaska, Belarus, Bulgaria, China, India, Iran, Israel, Italy, Mexico, Red, Russia, Spain, Ukraine, Yellowstone	4 breeds (n=5)<br>great Dane, mastiff, Saint Bernard, Shih Tzu	4 breeds (n=5)<br>dachshunds , Italian greyhound, miniature poodle, Yorkshire terrier	

Table S2 - Dog-derived SNPs and sequence discovered SNPs and indels.

SNPlex	Allele1	Allele2	SNPlex	Allele1	Allele2	Sequence	Allele1	Allele2
43199781	C	T	44269183	C	T	44226324	G	A
43200020	A	G	44278140	C	T	44226684	C	T
43200053	C	T	44278880	C	T	44226762	C	G
43200091	A	G	44280625	A	G	44226947	T	C
43471190	C	T	44281633	T	G	44227017	A	G
43471401	C	T	44282040	C	T	44227036	T	A
43471564	A	G	44284186	A	G	44227271	A	C
43627345	C	T	44285618	A	G	44227558	A	G
43708329	C	T	44285658	A	G	44227685	A	C
43712536	A	C	44292534	C	T	44227708	A	G
43767876	C	G	44300179	C	T	44228010	SINEC_CF	*
43771302	C	G	44320180	A	G	44228468	G	A
43811962	C	T	44346948	C	T	44228480	G	A
43812901	C	T	44348436	C	T	44228745	C	T
43812962	C	G	44349100	A	G	44228815	G	T
43961201	C	G	44349363	A	G	44230426	T	*
44108838	A	G	44349542	C	G	44229704	T	A
44127769	A	G	44350759	C	T	44230426	A	G
44134426	C	T	44353046	C	T	44230436	A	*
44184120	C	T	44357667	C	T	44230524	G	A
44195931	T	G	44393461	A	G	44230920	C	T
44195963	A	G	44512641	A	G	44231095	G	T
44200160	A	G	44518809	C	T	44231203	C	T
44202877	A	G	44537921	C	G	44231222	A	*
44203214	A	G	44541621	C	T	44231256	T	A
44204307	A	G	44550530	C	T	44231344	G	C
44209414	A	G	44565502	A	G	44231373	C	T
44209812	C	T	44568300	A	G	44231412	A	G
44212591	C	T	44568391	A	T	44231435	C	A
44212792	C	T	44569764	A	G	44231476	T	C
44218314	C	T	44586496	T	G	44232352	T	C
44226324	A	G	44594637	A	T	44232460	T	G
44226684	C	T	44597038	C	T	44232584	A	G
44236627	A	G	44669781	A	G	44232955	C	A
44236768	A	G	44697626	C	T	44234671	A	G

44237985	A	G	44698699	A	G	44234755	G	A
44238540	A	G	44708483	C	T	44235023	C	G
44239577	C	T	44736138	A	G	44235098	G	A
44249353	A	G	44835487	C	T			
44253636	C	T	44835614	C	T			
44258017	C	T	44841240	C	G			
44259236	C	T	44989978	A	T			
44260949	A	G	45337787	A	G			
44261639	A	T	45505902	C	G			
44261848	C	G	45716651	A	G			
44264050	C	T	46952832	C	T			
44265060	A	C	46952945	C	T			

Positions are from CanFam 1 assembly.

Table S3 - Sequenced amplicons across intron 2 of IGF1.

Amplicon	start	finish	4811bps	6331bps
5708	44226195	44226811	x	x
6014	44226947	44227475		x
6015	44227426	44228008	x	x
5709	44227973	44228525	x	x
5710	44228377	44228937	x	x
6016	44228930	44229431		x
6017	44229338	44229770		x
6018	44230426	44230762		x
6019	44230762	44231476	x	x
6020	44232323	44232998	x	x
6021	44232874	44233543		x
5996	44234649	44235185	x	x

Positions are from CanFam1 assembly.
