# Supplementary material for: The IGF1 small dog haplotype is derived from Middle Eastern grey wolves
Source: BMC Biol. 2010 Feb 24;8:16. doi: 10.1186/1741-7007-8-16 (PMC2837629; doi:10.1186/1741-7007-8-16)
Supplement: Additional file 2 — Dog-derived single nucleotide polymorphism (SNP) marker haplotypes based on 20 SNPs [15]. Counts are number of chromosomes. Greyed cells are the derived allele determined from golden jackal sequences. Bold haplotypes are small dog haplotypes. Italicized haplotypes are those with the least number of differences from small dog haplotype B. [file 1741-7007-8-16-S2.PDF]

44212792	44218314	44226324	44226684	44228468	44235098	44236627	44236768	44237388	44237985	44238540	44239577	44253636	44258017	44259236	44260949	44261639	44261848	44269183	44278140	IGF1 Dog Haplotypes*	Haplotype	Coyote	Alaska	Yellowstone	Banff	Isle Royale	Northern Quebec	Italy	Spain	Sweden	India	Israel	China	Total	Different from Hapotype B (small dog)
T	T	A	T	G	G	A	A	G	A	G	C	T	T	C	G	A	C	C	C	I	1	.	.	.	.	.	.	.	.	.	.	.	.	0	14
C	C	G	T	G	G	A	G	A	A	G	C	C	C	C	G	T	G	T	T	F	2	.	.	.	.	.	.	.	.	.	1	.	.	1	8
C	C	G	C	A	G	A	G	G	A	G	C	C	C	C	G	T	G	T	T	C	3	.	.	.	.	.	.	.	.	.	.	.	.	0	7
C	C	G	C	A	A	G	A	A	G	G	T	C	C	T	A	T	G	C	T	B	4	.	.	.	.	.	.	.	.	.	.	.	.	0	.
T	C	A	T			A	A		G	G	C	C	C	C	G	T	G	C	C		5	.	13	6	2	.	.	.	.	.	.	.	.	21	8
T	C	A	T			A	G		G	G	C	C	C	C	G	T	G	C	C		6	.	5	3	2	.	.	.	.	.	.	.	.	10	9
T	C	A	T			A	G		A	G	C	C	C	C	G	T	G	C	C		7	.	7	2	1	.	4	.	.	.	.	.	.	14	10
C	C	G	T			A	G		A	G	C	T	C	C	G	T	G	T	T		8	.	3	2	2	4	.	.	4	.	.	.	.	15	9
C	C	G	T			A	A		G	G	C	C	C	C	G	T	G	C	T		9	.	.	.	1	.	.	.	2	.	.	5	.	8	5
C	C	G	C			A	A		G	G	C	T	C	C	G	T	G	C	T		10	.	.	.	1	.	.	.	.	.	.	.	.	1	5
C	C	A	T			A	A		G	G	C	C	C	C	G	T	G	C	C		11	.	10	9	.	.	17	.	10	.	.	.	.	46	7
C	C	G	T			A	G		G	G	C	T	C	C	G	T	G	C	T		12	.	.	1	.	.	.	.	.	.	.	.	.	1	7
C	C	G	C			A	G		G	G	C	C	C	C	G	T	G	C	T		13	18	.	1	.	.	.	.	.	.	.	.	.	19	5
C	C	G	C			A	G		G	G	T	C	C	C	G	T	G	T	T		14	1	.	.	.	.	.	.	.	.	.	.	.	1	5
C	C	G	T			A	G		G	G	C	C	C	C	G	T	G	C	C		15	.	.	.	2	7	1	.	.	11	1	.	.	22	7
C	C	G	C			A	G		A	G	C	C	C	C	G	T	G	C	T		16	1	.	.	.	.	.	.	.	.	.	.	.	1	6
C	C	G	T			A	G		A	G	C	C	C	C	G	T	G	C	C		17	.	.	.	.	1	4	.	2	.	.	.	.	7	8
T	C	A	T			A	A		G	G	C	C	C	C	A	T	G	C	C		18	.	1	.	.	.	.	.	.	.	.	.	.	1	7
C	C	G	T			A	G		G	G	C	C	C	C	G	T	G	T	C		19	.	.	.	.	1	.	.	.	.	.	.	.	1	8
C	C	G	T			A	G		A	G	C	C	C	C	G	T	G	T	C		20	1	.	.	.	6	.	.	1	.	.	.	.	8	9
T	C	G	T			A	G		A	G	C	C	C	C	G	T	G	C	T		21	.	.	.	.	1	.	.	.	.	.	.	6	7	8
C	C	G	C			A	A		G	G	C	C	C	C	A	T	G	C	T		22	.	.	.	.	.	.	.	1	.	.	.	.	1	3
C	C	G	T			A	A		G	G	T	T	C	C	G	T	G	T	T		23	.	.	.	.	.	.	.	2	.	.	.	.	2	6
C	C	G	C			A	A		G	G	T	C	C	C	G	T	G	T	C		24	.	.	.	.	.	.	.	8	.	.	1	.	9	5
C	C	A	T			A	G		A	G	C	C	C	C	G	T	G	T	C		25	1	.	.	.	.	.	.	2	.	.	.	.	3	10
C	C	G	T			A	A		G	G	C	C	C	C	G	T	G	C	C		26	.	.	.	.	.	.	.	7	.	.	1	.	8	6
C	C	A	T			A	G		A	G	C	C	C	C	G	T	G	C	C		27	.	.	.	.	.	.	.	1	.	.	.	.	1	9
C	C	G	T			A	A		A	G	C	C	C	T	G	T	G	T	C		28	.	.	.	.	.	.	.	1	.	.	.	.	1	7
C	C	G	T			A	A		G	G	C	C	C	C	A	T	G	C	T		29	.	.	.	.	.	.	.	1	.	.	.	.	1	4
C	C	A	T			A	A		G	G	T	C	C	C	G	T	G	T	C		30	.	.	.	.	.	.	.	2	.	.	.	.	2	7
C	C	G	T			A	G		A	G	C	C	C	C	G	T	G	C	T		31	.	.	.	1	.	.	.	4	.	.	1	.	6	7
T	C	G	T			A	A		G	G	C	C	C	C	G	T	G	C	C		32	.	.	.	.	.	.	.	.	.	.	.	2	2	7
C	C	G	T			A	A		G	G	C	C	C	C	G	T	G	T	T		33	.	.	.	.	.	.	.	.	.	.	1	.	1	6
C	C	G	T			A	G		A	G	C	T	C	C	G	T	G	C	T		34	.	.	1	.	.	.	.	.	8	.	.	.	9	8
C	C	G	T			A	G		A	G	C	C	T	C	G	T	G	T	T		35	.	.	.	.	.	.	.	.	6	.	.	.	6	9
C	C	G	T			A	A		A	G	C	C	C	T	A	T	G	T	T		36	.	.	.	.	.	.	.	.	8	.	.	.	8	5
C	C	G	T			A	G		A	G	C	T	T	C	G	A	C	C	C		37	.	.	.	.	.	.	2	.	.	.	.	.	2	12
C	C	G	C			A	A		G	G	C	C	C	C	G	T	G	T	T		38	.	.	.	.	.	.	.	.	.	4	.	.	4	5
C	C	G	C			A	G		G	G	C	C	C	C	G	T	G	C	C		39	5	.	.	.	.	.	.	.	.	.	.	.	5	6
C	C	G	C			A	G		G	G	C	C	C	C	G	T	G	T	C		40	5	.	.	.	.	.	.	.	.	.	.	.	5	7
C	C	G	C			A	G		A	G	C	C	C	C	G	T	G	T	C		41	6	.	.	.	.	.	.	.	.	.	.	.	6	8
C	C	G	C			A	G		G	G	C	C	C	C	G	T	G	T	T		42	3	.	.	.	.	.	.	.	.	.	.	.	3	6
C	T	G	T			A	G		G	G	C	T	C	C	G	T	G	C	T		43	.	.	1	.	.	.	.	.	.	.	.	.	1	8
C	C	G	C			A	G		A	G	C	C	C	C	G	T	G	C	C		44	1	1	.	.	.	.	.	.	.	.	.	.	2	7
C	C	G	T			A	A		G	G	C	T	C	C	G	T	G	C	T		45	.	.	.	.	.	.	.	1	.	.	.	.	1	6
C	C	G	C			A	A		G	G	C	C	C	C	G	T	G	C	C		46	.	.	.	.	.	.	.	1	.	.	.	.	1	5
C	C	G	C			A	A		G	G	C	C	C	C	G	T	G	C	T		47	.	.	.	.	.	.	.	1	.	.	.	.	1	4
C	C	G	C			A	A		G	G	T	C	C	T	G	T	G	T	C		48	.	.	.	.	.	.	.	1	.	.	.	.	1	4
C	C	A	C			A	A		A	G	C	C	C	T	G	T	G	T	C		49	.	.	.	.	.	.	.	1	.	.	.	.	1	7
C	C	G	T			A	A		G	G	T	C	C	C	A	T	G	T	T		50	.	.	.	.	.	.	.	1	.	.	.	.	1	4
C	C	G	T			A	A		G	G	T	C	C	C	G	T	G	C	T		51	.	.	.	.	.	.	.	.	.	.	1	.	1	4
C	T	G	T			A	G		A	G	C	C	T	T	G	T	G	T	T		52	.	.	.	.	.	.	.	.	1	.	.	.	1	9
																						42	40	26	12	20	26	2	54	34	6	10	8	280	

* See Sutter et al. 2007
