# Supplementary material for: The IGF1 small dog haplotype is derived from Middle Eastern grey wolves
Source: BMC Biol. 2010 Feb 24;8:16. doi: 10.1186/1741-7007-8-16 (PMC2837629; doi:10.1186/1741-7007-8-16)
Supplement: Additional file 3 — Single nucleotide polymorphism haplotypes from sequenced regions of intron 2 (Figure 1). Counts are number of chromosomes. Grey vertical bar indicates a recombination point. Greyed cells are the derived allele determine from ancestral golden jackal sequences. Bold haplotypes are small dog haplotypes. [file 1741-7007-8-16-S3.PDF]

Sequence	SNP CanFam1 Position																																						Haplotype
	44226324	44226684	44226762	44226947	44227017	44227036	44227271	44227558	44227685	44227708	44228010	44228468	44228480	44228745	44228815	44229590	44229704	44230426	44230436	44230524	44230920	44231095	44231203	44231222	44231256	44231344	44231373	44231412	44231435	44231476	44232352	44232460	44232584	44232955	44234671	44234755	44235023	44235098	
6331 bps	G	C	C	T	A	T	A	A		A	----	G	G	C	G	T	T	A	A	G		G		-	T	G	C	A	C	T	T	T	A	C	A	G	C	G	01
	G	C	C	C	A	A	C	A		A	----	G	A	T	G	T	A	A	A	G		G		A	T	G	T	A	C	C	T	G	A	C	G	G	C	G	02
	G	C	C	C	A	A	C	A		A	SINE	A	G	T	G	T	A	A	A	G		T		A	T	G	C	G	C	C	C	T	A	A	G	G	C	G	03
	G	T	C	C	A	A	C	A		A	----	G	G	T	G	T	A	A	A	G		T		A	T	G	C	G	C	C	C	T	A	A	G	G	C	G	04
	G	C	C	C	A	A	C	A		A	SINE	A	G	T	G	T	A	A	A	G		G		A	T	G	C	A	C	C	C	T	A	C	G	A	C	A	05
	A	T	C	C	A	A	C	A		A	----	G	G	T	G	T	A	A	A	G		T		A	T	G	C	G	C	C	C	T	A	A	G	G	C	G	06
	A	T	C	C	A	A	C	A		A	----	G	G	T	G	T	A	A	A	A		T		A	T	G	C	G	C	C	C	T	A	A	G	G	C	G	07
	G	T	G	C	G	A	C	A		A	----	G	G	T	G	T	A	A	A	G		T		A	A	C	C	G	C	C	C	T	G	A	G	G	G	G	08
	G	T	C	C	A	A	C	A		A	----	G	G	T	G	T	A	A	A	G		G		A	T	G	C	A	A	C	C	T	A	C	G	G	C	G	09
	A	T	C	C	A	A	C	A		A	----	G	G	T	G	T	A	A	-	G		T		A	T	G	C	G	C	C	C	T	A	A	G	G	C	G	10
	G	C	C	C	A	A	C	A		G	----	G	G	T	G	T	A	A	A	G		G		A	T	G	C	A	A	C	C	T	A	C	G	A	C	A	11
	A	T	C	C	A	A	C	A		A	----	G	G	T	G	-	A	G	A	G		T		A	T	G	C	G	C	C	C	T	A	A	G	G	C	G	12
	G	C	C	C	A	A	C	A		A	----	G	G	T	G	T	A	A	A	G		G		A	T	G	C	A	A	C	C	T	A	C	G	A	C	A	13
	G	C	C	C	A	A	C	G		A	----	G	G	T	T	T	A	A	A	G		G		A	T	G	C	A	A	C	C	T	A	C	G	A	C	A	14
																																							Total
4811 bps	G	C	C					A	A	A		G	G	C	G			A	A	G	C	G	C	-	T	G	C	A	C	T	T	T	A	C	A	G	C	G	01
	G	C	C					A	A	A		G	A	T	G			A	A	G	T	G	C	A	T	G	T	A	C	C	T	G	A	C	G	G	C	G	02
	G	C	C					A	A	A		A	G	T	G			A	A	G	T	G	C	A	T	G	C	A	C	C	C	T	A	C	G	A	C	A	03
	G	C	C					A	A	A		A	G	T	G			A	A	G	T	T	C	A	T	G	C	G	C	C	C	T	A	A	G	G	C	G	04
	G	T	C					A	A	A		G	G	T	G			A	A	G	T	T	C	A	T	G	C	G	C	C	C	T	A	A	G	G	C	G	05
	G	T	C					A	A	A		G	G	T	G			A	A	A	T	T	C	A	T	G	C	G	C	C	C	T	A	A	G	G	C	G	06
	A	T	C					A	A	A		G	G	T	G			A	A	G	T	T	C	A	T	G	C	G	C	C	C	T	A	A	G	G	C	G	07
	A	T	C					A	A	A		G	G	T	G			A	A	A	T	T	C	A	T	G	C	G	C	C	C	T	A	A	G	G	C	G	08
	A	T	C					A	A	A		G	G	T	G			A	-	G	T	T	C	A	T	G	C	G	C	C	C	T	A	A	G	G	C	G	09
	A	T	C					A	A	A		G	G	T	G			G	A	G	T	T	C	A	T	G	C	G	C	C	C	T	A	A	G	G	C	G	10
	A	T	C					A	C	A		G	G	T	G			A	A	G	T	T	C	A	T	G	C	G	C	C	C	T	A	A	G	G	C	G	11
	G	T	G					A	A	A		G	G	T	G			A	A	G	T	T	C	A	A	C	C	G	C	C	C	T	G	A	G	G	G	G	12
	G	C	C					A	A	A		G	G	T	G			A	A	G	T	T	C	A	T	G	C	G	C	C	T	G	A	C	G	G	C	G	13
	G	T	C					A	A	A		G	G	T	G			A	A	G	T	T	C	A	T	G	C	G	C	C	C	T	A	A	G	A	C	A	14
	G	T	C					A	A	A		G	G	T	G			A	A	G	T	G	C	A	T	G	C	A	A	C	C	T	A	C	G	G	C	G	15
	G	T	C					A	C	A		G	G	T	G			A	A	G	T	T	C	A	T	G	C	G	C	C	C	T	A	A	G	G	C	G	16
	G	C	C					A	A	A		G	G	T	G			A	A	G	T	G	C	A	T	G	C	A	C	C	C	T	A	C	G	A	C	A	17
	G	C	C					A	A	A		G	G	T	G			A	A	G	T	G	C	A	T	G	C	A	A	C	C	T	A	C	G	A	C	A	18
	G	C	C					A	A	A		G	G	T	G			A	-	G	T	G	T	A	T	G	C	A	C	C	C	T	A	C	G	G	C	G	19
	G	C	C					G	A	G		G	G	T	G			A	A	G	T	G	C	A	T	G	C	A	C	C	C	T	A	C	G	A	C	A	20
	G	C	C					G	A	G		G	G	T	T			A	A	G	T	G	C	A	T	G	C	A	C	C	C	T	A	C	G	A	C	A	21
																																							Total

Sequence	Haplotype	Small Dogs				Giant Dogs				New World Wolves				Old World Wolves											
		Yorkshire Terrier	Italian Greyhound	Miniature Poodle	Dachshund	Shih Tzu	Great Dane	Mastiff	Saint Bernard	Alaska	Mexican	Red	Yellowstone	Belarus	Bulgaria	China	India	Iran	Israel	Italy	Russia	Spain	Ukraine	Coyote	Total Chromosomes
6331 bps	01	.	.	.	.	.	.	.	.	.	.	.	.	.	.	.	.	.	.	.	.	.	.	2	2
	02	.	.	.	.	.	.	.	.	.	.	.	.	.	.	.	.	.	.	.	.	.	.	2	2
	03	.	2	2	.	.	.	.	.	.	.	.	.	.	.	.	.	.	.	.	.	.	.	.	4
	04	.	.	.	.	.	2	.	2	.	.	.	.	.	.	2	.	.	.	4	.	.	.	.	10
	05	2	.	.	.	.	.	.	.	.	.	.	.	.	.	.	.	.	.	.	.	.	.	.	2
	06	.	.	.	.	2	.	2	.	2	.	.	3	.	.	.	.	.	.	.	.	.	.	.	9
	07	.	.	.	.	.	.	.	2	.	.	.	.	.	.	.	.	.	.	.	.	.	.	.	2
	08	.	.	.	.	.	.	.	.	.	.	.	.	.	.	.	.	.	.	.	.	2	.	.	2
	09	.	.	.	.	.	.	.	.	.	.	.	.	.	.	.	.	.	1	.	.	.	.	.	1
	10	.	.	.	.	.	.	.	.	.	.	.	.	.	.	.	.	.	.	.	.	2	.	.	2
	11	.	.	.	.	.	.	.	.	.	.	.	.	.	.	.	.	.	1	.	.	.	.	.	1
	12	.	.	.	.	.	.	.	.	.	.	.	1	.	.	.	.	.	.	.	.	.	.	.	1
	13	.	.	.	.	.	.	.	.	.	.	.	.	.	.	.	.	.	1	.	.	.	.	.	1
	14	.	.	.	.	.	.	.	.	.	.	.	.	.	.	.	.	.	1	.	.	.	.	.	1
	Total	2	2	2	0	2	2	2	4	2	0	0	4	0	0	2	0	0	4	4	0	4	0	4	40
4811 bps	01	.	.	.	.	.	.	.	.	.	.	.	.	.	.	.	.	.	.	.	.	.	.	2	2
	02	.	.	.	.	.	.	.	.	.	.	.	.	.	.	.	.	.	.	.	.	.	.	2	2
	03	2	.	.	1	.	.	.	.	.	.	.	.	.	.	.	.	.	.	.	.	.	.	.	3
	04	.	2	2	.	.	.	.	.	.	.	.	.	.	.	.	.	.	.	.	.	.	.	.	4
	05	.	.	.	.	.	2	.	.	1	.	.	.	2	1	3	1	1	5	4	.	.	2	.	22
	06	.	.	.	.	.	.	.	2	.	.	.	.	.	.	.	.	.	.	.	.	.	.	.	2
	07	.	.	.	3	2	.	2	.	1	2	.	3	.	.	1	.	.	.	.	1	.	.	.	15
	08	.	.	.	.	.	.	.	2	.	.	.	.	.	.	.	.	.	.	.	.	.	.	.	2
	09	.	.	.	.	.	.	.	.	1	.	.	.	.	.	.	.	.	.	.	.	2	.	.	3
	10	.	.	.	.	.	.	.	.	.	.	.	1	.	.	.	.	.	.	.	.	.	.	.	1
	11	.	.	.	.	.	.	.	.	1	2	.	.	.	.	.	.	.	.	.	.	.	.	.	3
	12	.	.	.	.	.	.	.	.	.	.	.	.	.	.	.	.	.	.	.	.	2	.	.	2
	13	.	.	.	.	.	.	.	.	.	.	2	.	.	.	.	.	.	.	.	.	.	.	.	2
	14	.	.	.	.	.	.	.	.	.	.	.	.	.	1	.	.	.	.	.	.	.	.	.	1
	15	.	.	.	.	.	.	.	.	.	.	.	.	.	.	.	.	.	1	.	.	.	.	.	1
	16	.	.	.	.	.	.	.	.	.	.	.	.	.	.	.	.	.	1	.	1	.	.	.	2
	17	.	.	.	.	.	.	.	.	.	.	.	.	.	.	.	.	.	2	.	.	.	.	.	2
	18	.	.	.	.	.	.	.	.	.	.	.	.	.	.	.	.	.	1	.	.	.	.	.	1
	19	.	.	.	.	.	.	.	.	.	.	.	.	.	.	.	.	.	1	.	.	.	.	.	1
	20	.	.	.	.	.	.	.	.	.	.	.	.	.	.	.	3	1	3	.	.	.	.	.	7
	21	.	.	.	.	.	.	.	.	.	.	.	.	.	.	.	.	.	2	.	.	.	.	.	2
	Total	2	2	2	4	2	2	2	4	4	4	2	4	2	2	4	4	2	16	4	2	4	2	4	80
