# Supplementary material for: The IGF1 small dog haplotype is derived from Middle Eastern grey wolves
Source: BMC Biol. 2010 Feb 24;8:16. doi: 10.1186/1741-7007-8-16 (PMC2837629; doi:10.1186/1741-7007-8-16)
Supplement: Additional file 4 — Matrix of single nucleotide polymorphism differences between haplotypes from 4811 bp of sequence. Bold haplotypes indicate small dog haplotypes. Bold numbers indicate haplotypes with smallest number of differences to the common small dog haplotype B. [file 1741-7007-8-16-S4.PDF]

| | Hap1 | Hap2 | **Hap3 (HapB)** | **Hap4 (HapC)** | Hap5 | Hap6 | Hap7 | Hap8 | Hap9 | Hap10 | Hap11 | Hap12 | Hap13 | Hap14 | Hap15 | Hap16 | Hap17 (Israel) | Hap18 (Israel) | Hap19 | Hap20 (Israel, Iran, India) | Hap21 (Israel) |
|---|---|---|---|---|---|---|---|---|---|---|---|---|---|---|---|---|---|---|---|---|---|
| Hap1 | | | | | | | | | | | | | | | | | | | | | |
| Hap2 | 8 | | | | | | | | | | | | | | | | | | | | |
| **Hap3 (HapB)** | 9 | 7 | | | | | | | | | | | | | | | | | | | |
| **Hap4 (HapC)** | 10 | 8 | 5 | | | | | | | | | | | | | | | | | | |
| Hap5 | 10 | 8 | 7 | 2 | | | | | | | | | | | | | | | | | |
| Hap6 | 11 | 9 | 8 | 3 | 1 | | | | | | | | | | | | | | | | |
| Hap7 | 11 | 9 | 8 | 3 | 1 | 2 | | | | | | | | | | | | | | | |
| Hap8 | 12 | 10 | 9 | 4 | 2 | 1 | 1 | | | | | | | | | | | | | | |
| Hap9 | 12 | 10 | 9 | 4 | 2 | 3 | 1 | 2 | | | | | | | | | | | | | |
| Hap10 | 12 | 10 | 9 | 4 | 2 | 3 | 1 | 2 | 2 | | | | | | | | | | | | |
| Hap11 | 12 | 10 | 9 | 4 | 2 | 3 | 1 | 2 | 2 | 2 | | | | | | | | | | | |
| Hap12 | 15 | 13 | 12 | 7 | 5 | 6 | 6 | 7 | 7 | 7 | 7 | | | | | | | | | | |
| Hap13 | 8 | 4 | 7 | 4 | 4 | 5 | 5 | 6 | 6 | 6 | 6 | 9 | | | | | | | | | |
| Hap14 | 12 | 10 | 5 | 4 | 2 | 3 | 3 | 4 | 4 | 4 | 4 | 7 | 6 | | | | | | | | |
| Hap15 | 8 | 6 | 5 | 6 | 4 | 5 | 5 | 6 | 6 | 6 | 6 | 9 | 6 | 6 | | | | | | | |
| Hap16 | 11 | 9 | 8 | 3 | 1 | 2 | 2 | 3 | 3 | 3 | 1 | 6 | 5 | 3 | 5 | | | | | | |
| Hap17 (Israel) | 8 | 6 | **1** | 6 | 6 | 7 | 7 | 8 | 8 | 8 | 8 | 11 | 6 | 4 | 4 | 7 | | | | | |
| Hap18 (Israel) | 9 | 7 | **2** | 7 | 7 | 8 | 8 | 9 | 9 | 9 | 9 | 12 | 7 | 5 | 3 | 8 | 1 | | | | |
| Hap19 | 8 | 6 | 5 | 6 | 6 | 7 | 7 | 8 | 6 | 8 | 8 | 11 | 6 | 8 | 4 | 7 | 4 | 5 | | | |
| Hap20 (Israel, Iran, India) | 10 | 8 | **3** | 8 | 8 | 9 | 9 | 10 | 10 | 10 | 10 | 13 | 8 | 6 | 6 | 9 | 2 | 3 | 6 | | |
| Hap21 (Israel) | 11 | 9 | **4** | 9 | 9 | 10 | 10 | 11 | 11 | 11 | 11 | 14 | 9 | 7 | 7 | 10 | 3 | 4 | 7 | 1 | |
